# Impact of consumer confidence on the expected returns of the Tokyo Stock Exchange: A comparative analysis of consumption and production-based asset pricing models

**DOI:** 10.1371/journal.pone.0241318

**Published:** 2020-11-03

**Authors:** Javier Rojo-Suárez, Ana Belén Alonso-Conde

**Affiliations:** Department of Business Administration, Rey Juan Carlos University, Madrid, Spain; Sam Houston State University, UNITED STATES

## Abstract

Most single-factor and multifactor asset pricing models constitute special cases of the consumption-based asset pricing theory, in which investors’ marginal utility is the key determinant of asset prices. However, in recent years, production-based asset pricing models have been extraordinarily successful in correctly pricing a wide range of anomaly portfolios that are typically mispriced in previous research. In parallel, research on conditioning information has contributed to significantly improve the performance of classic consumption-based asset pricing models. On this basis, in this paper we conduct an in-depth research on the performance of consumption and production-based asset pricing models on the Tokyo Stock Exchange, for the period from 1992 to 2018, in order to test to what extent consumer confidence helps consumption models to correctly capture shifts in the investment opportunity set of investors. To overcome the constraints imposed by the periodicity of macroeconomic data, we use a factor-mimicking portfolio approach that allows us to test the performance of the models into consideration at different frequencies. Our results suggest that the consumer confidence index for Japan helps consumption-based asset pricing models outperform production-based models for different anomaly portfolios. Conversely, in those cases where consumption models perform worse, the production models also perform poorly. These results help to partially reconcile the results provided by the consumption and production models, and constitute a step forward for the purpose of identifying the fundamental risk factors that drive asset prices.

## Introduction

The asset pricing theory comprises a wide range of models and methodologies that seek to find the fundamental risk factors that drive asset prices and expected returns. A large part of the asset pricing literature focuses on explaining the cross-sectional behavior of stock returns. In particular, most cross-sectional asset pricing models are intended to identify the key factors that make some stocks provide higher expected returns than others. This task is crucial in a wide range of well-differentiated areas, such as corporate finance, accounting, asset portfolio management, or fiscal and monetary policy [1].

Considering that the vast majority of asset pricing models are special cases of consumption-based or production-based models, this paper pursues two main objectives. First, building on the fact that most of the recent evidence on the performance of consumption-based asset pricing models attributes their poor explanatory power to their failure to correctly capture conditioning information [2, 3], this paper seeks to determine to what extent the consumer sentiment, and more particularly the consumer confidence index (hereafter, CCI), allows the capital asset pricing model (hereafter, CAPM) and the consumption-CAPM (hereafter, CCAPM) to improve their performance on the Tokyo Stock Exchange. The specific characteristics of the Japanese economy due to its demographic conditions, as well as its consumption pattern over time, largely motivate our study. According to [4], the steady increase in the ratio of the population older than 64 to the population between 15 and 64 since 1990 is strongly related to the decreases in inflation and the output of Japan in the same period. On the other hand [5], relate the increase in the number of retirees to the sales of Japanese stocks and bonds in recent decades and the increase in foreign investments, which ultimately results in yen appreciation and deflation. We believe that these elements can result in specific patterns in the relationship between consumption growth and consumer sentiment, and this paper explores their effect on the returns of the Tokyo Stock Exchange.

Second, this paper seeks to determine the prevalence of the successful results achieved by recent production models, specifically the model proposed by [6] and [7] (hereafter, the Fama-French five-factor model and the *q*-model, respectively), in equity markets other than those studied by these authors (i.e. US stock markets). More particularly, we seek to study the apparent redundancy of the *value factor*, used as an explanatory variable in the Fama-French five-factor model, in the presence of investment factors. For that purpose, we compare the performance of the Fama-French five-factor model with that of the *q*-model, on the Tokyo Stock Exchange, for the period from 1992 to 2018, at different frequencies and using distinct anomaly portfolios as test assets.

This paper contributes to the literature that relates consumer sentiment with asset prices, and to the literature that seeks to reconcile both consumption-based and production-based asset pricing models to integrate both approaches into a more general equilibrium model. To the best of our knowledge, this is the first study to compare the performance of consumption and production models on the Tokyo Stock Exchange, using the CCI as an instrument to incorporate conditioning information into consumption models.

Our results show that the CCI does an excellent job when used as an instrument in consumption-based asset pricing models, helping them to greatly improve their performance and outperform production models for some portfolios. Additionally, our results show that, although the Fama-French five-factor model and the *q*-model provide the lowest pricing errors and the highest *R*^2^ statistics among all the models under study, in most cases the Fama-French five-factor model is the best-performing model, followed by the *q*-model. These results suggest that, while there may be some duplication in the explanatory power of the value factor and that of investment factors, they do not appear to be completely redundant.

Hereafter, the paper proceeds as follows. The next section describes the theoretical background of the models under consideration. The section thereafter defines the models and methods under analysis. This is followed by a section that describes the data and the results. The last section discusses the results and concludes the paper.

### Theoretical background

A large number of asset pricing models extensively studied in the literature are special cases of a general consumption-based asset pricing model, in which the marginal utility of investors is the only factor that drives asset prices [8] (pp.149-183). Specifically, some prominent models, such as the CAPM [9–11], the CCAPM [12, 13], or the Fama-French three-factor model [14], are developed by naturally imposing some constraint on the investors’ utility function or the dynamics of consumption growth. In this regard, the Fama-French three-factor model deserves special mention. According to [15], the Fama-French three-factor model constitutes a successful implementation of the intertemporal asset pricing theory, as defined by [16]. On this basis, the wealth portfolio–frequently assimilated to a broad-based portfolio, such as the value-weighted portfolio of the Tokyo Stock Exchange–does not correctly proxy the marginal utility of investors, given the forecastable nature of expected returns. This makes it necessary to introduce other state variables that allow the model to forecast shifts in the investment opportunity set of investors [8] (pp. 165–167). The Fama-French three-factor model introduces two additional state variables apart from the value-weighted market portfolio, specifically a size factor and a value factor, that allow the model to correctly price a wide range of anomaly portfolios. These factors are often referred to as SMB (the excess return of the portfolio that comprises the small minus big market value firms) and HML (the excess return of the portfolio that comprises the high minus low book-to-market equity firms) in the asset pricing literature.

Although consumption-based asset pricing models are generally presented in the form of single-factor or multifactor models, they can all be written in terms of a stochastic discount factor (hereafter, SDF) model. This model assumes that asset prices are determined by the expectation conditional on time-*t* information of the product of the SDF and the asset payoff, where the SDF is the marginal rate of substitution or ratio of price to probability for all states of nature [8] (pp. 6–7). The SDF model is the dominant approach in contemporary asset pricing research [17] (p. 83), as it is easily adaptable to most of the topics addressed in the asset pricing literature. Below we exploit this flexibility to allow consumption-based asset pricing models to account for conditioning information used by economic agents and, more particularly, consumer sentiment.

Although recent research on financial markets highlights the important implications of consumer sentiment on asset returns [18–21] and its strong predictive power [22–25], its role in the cross-sectional analysis of stock returns has not yet been sufficiently addressed [26]. evaluates the validity of the Arbitrage Pricing Theory (hereafter, APT) in the Japanese equity market using investor confidence as an explanatory variable for asset returns, among other factors. Although his results support evidence of the APT as opposed to the CAPM, the author proxies investor confidence by the difference between the returns of long-term corporate and government bonds, using data series that cover only ten years (from January 1975 to December 1984) [27]. use an APT model to study the extent to which different macroeconomic factors determine returns in the Japanese stock market. Although the authors ignore investor confidence into the model, they use several variables frequently used as a proxy for consumer sentiment. Their results show that money supply, inflation, exchange rate, and industrial production have a significant influence on expected returns.

On the other hand [28], studies how market indicators of investor sentiment and arbitrage constraints affect the performance of cross-country stock market anomalies. His results show that most anomaly-based strategies are poorly related to market sentiment except value strategies. Remarkably, the author uses a composite measure of market sentiment that comprises the market-level investor sentiment index suggested by [29], the State Street Investor Confidence Index (SSIC), the Sentix Economic Indices Global Aggregate Overall Index (Sentix), and the Weighted Manufacturing and Non-Manufacturing Composite Purchasing Managers’ Index (PMI).

In contrast, we use the CCI for Japan, as provided by the OECD, within the ‘Composite Leading Indicators’ dataset, as a proxy for consumer sentiment. This data series comprises a large number of components, including real economy- and credit market-oriented indicators, which make the CCI a useful instrument to capture conditioning information used by investors, as noted below. However, it should be noted that many other variables can be used for the same purpose, given their strong relationship with business cycle. This is the case of illiquidity measures [30, 31] or market volatility, as measured by indicators such as the Chicago Board Options Exchange Market Volatility Index (VIX) [32–34].

Notwithstanding the above, consumption-based asset pricing models are only a partial explanation for asset prices, since they focus exclusively on the demand-side of the economy. Conversely, production models focus on the supply-side, seeking to connect asset prices to firms’ profitability and investment. Although first production models are long-standing [35–40], recent research has considerably improved their performance, allowing some production models to correctly price a wide range of anomaly portfolios that are typically mispriced in previous research. As noted above, the Fama-French five-factor model and the *q*-model, developed by [6] and [7], respectively, are especially remarkable [6]. rely on the present value identity, as defined by [41], to add two investment-inspired factors to their three-factor model, namely, RMW (the excess return of the portfolio that comprises the most profitable stocks minus the least profitable) and CMA (the excess return of the portfolio that comprises firms that invest conservatively minus aggressively) [7]. adopt a similar approach to propose a four-factor model (i.e. the *q*-model) that explicitly ignores HML. Importantly, the *q*-model fits several anomalies better than other models that, as the [42] four-factor model, include a momentum factor (the excess return of the portfolio that comprises the high minus low past one-year return stocks) as an explanatory variable. In fact, the apparent convenience of including HML as a factor is currently the subject of a lively debate, to the point that [7] and [43] claim that HML and the momentum factor appear to be noisy versions of the *q*-factors.

## Models and methods

In this section we first briefly summarize the general framework of consumption-based asset pricing models and relate it to factor models, as they are the most widely-used representations in empirical research. Then, we describe the basic procedure that allows the model to account for the conditioning information by parameterizing the model coefficients. Additionally, we describe the factor-mimicking portfolio method, which allows us to switch from a factor-based asset pricing model to a SDF model by comprising all factors into a single explanatory variable (i.e. the factor-mimicking portfolio). This transformation is essential for some macroeconomic asset pricing models, given the presence of low-frequency series–e.g. consumption data–among the factors considered. Finally, we summarize the general framework of production models and illustrate the general mechanics of the production models under study, namely, the Fama-French five-factor model and the *q*-model.

### Consumption models

According to the Existence Theorem, the law of one price–that is, the fact that every asset that provides the same payoff as another has the same price–guarantees the existence of a single payoff x* in the payoff space that perfectly prices all payoffs [44–46]. In this context, the following pricing function is exactly satisfied:

$$p_{t}=E_{t}\left(x_{t+1}^{*}x_{t+1}\right)$$ (1)

where **p**_**t**_ is the *N*-dimensional vector of asset prices at time *t*, **E**_**t**_ (·) is the expectation conditional on time-*t* information, and **x**_**t**+**1**_ is the *N*-dimensional vector of asset payoffs. Since asset returns are investments with price one and payoff *R*_*t*+1_, Expression (1) can be particularized as follows:

$$E_{t}(x_{t+1}^{*}R_{t+1})=1_{N}$$ (2)

where **R**_**t+1**_ is the *N*-dimensional vector of gross returns. Defining an excess return as the difference between two returns, then:

$$E_{t}(x_{t+1}^{*}R_{t+1}^{e})=0_{N}$$ (3)

where $R_{t+1}^{e}$ is the *N*-dimensional vector of excess returns in the payoff space. Additionally to the law of one price, when the payoff space and the pricing function leave no arbitrage opportunities, at least one strictly positive SDF is guaranteed. If markets are complete, that is, if there are the same number of securities as states of nature, that ensures that $x_{t+1}^{*}$ is the only valid SDF and that it is positive in all states of nature. However, when markets are incomplete there are a potentially infinite number of SDFs that satisfy the pricing function and such positive SDF no longer needs to be in the payoff space. In that case, Expression (3) can be written as follows:

$$E_{t}(m_{t+1}R_{t+1}^{e})=0_{N}$$ (4)

where *m*_*t*+1_ denotes any strictly positive SDF. Since the expected utility theory assumes that marginal utility is always nonnegative, the Existence Theorem allows for a macroeconomic explanation of asset prices. In particular, the investor’s first-order condition allows us to write Expression (4) as follows [8] (pp. 4–5):

$$E_{t}\left[\frac{u^{\prime }\left(C_{t+1}\right)}{u^{\prime }\left(C_{t}\right)}R_{t+1}^{e}\right]=0_{N}$$ (5)

where *u*’ is the investor’s marginal utility and *C*_*t*_ denotes the consumption in levels at date *t*. Assuming that *m*_*t*+1_ is linear in consumption growth, the SDF can be written as follows (for a complete review of the procedures that allow linearizing the SDF in Expression (5) see [8] (pp. 161–165)):

$$m_{t+1}=a_{t}+b_{\text{t}}\Delta C_{t+1}$$ (6)

where Δ*C*_*t*+1_ denotes consumption growth. Expression (4) combined with Expression (6) results in the CCAPM. However, many consumption-based asset pricing models overcome measurement problems tied to consumption data using broad-based portfolios as a proxy for marginal utility growth. Specifically, different assumptions about investors’ preferences, such as quadratic utility, log utility, or the combination of exponential utility with normal distributions, make the return on the wealth portfolio a valid proxy for consumption growth [8] (pp. 152–161). In that framework, Expression (4) becomes the CAPM, with the wealth portfolio typically being assimilated to the value-weighted market portfolio minus the risk-free rate (hereafter, RMRF) in empirical research. On the other hand, the Fama-French three-factor model uses three different portfolios (RMRF, SMB and HML) as consumption state variables of special hedging concern to investors, in the language of the intertemporal CAPM, as defined by [16]. In order to allow Expression (4) to encompass all of these models, we can generalize Expression (6) as follows:

$$m_{t+1}=a_{t}+b_{t}^{'}f_{t+1}$$ (7)

where *a*_*t*_ and **b**_**t**_ are parameters and **f**_**t+1**_ is the *K*-dimensional vector of fundamental factors used as a proxy for the growth in marginal utility (e.g. the consumption growth for the CCAPM, RMRF for the CAPM, and RMRF, SMB and HML for the Fama-French three-factor model). However, it is important to note that, contrary to Expression (6), Expression (7) does not assume a specific economic structure in the SDF. This means that any vector of factors whose covariances with excess returns are perfectly correlated with expected excess returns will price assets correctly. Therefore, without imposing any other restriction on the dynamics of the vector of factors **f**_**t+1**_, Expression (7) is fully consistent with the mechanics of the APT [47, 48].

Although most empirical work on asset pricing uses beta models rather than the SDF model, the transformation of Expression (4) into a beta model is straightforward. Specifically, Expression (4) can be rearranged as follows:

$$E_{t}\left(R_{t+1}^{e}\right)=-\frac{cov_{t}\left(m_{t+1},R_{t+1}^{e}\right)}{E_{t}\left(m_{t+1}\right)}$$ (8)

or equivalently:

$$E_{t}\left(R_{t+1}^{e}\right)=\left[\frac{cov_{t}\left(m_{t+1},R_{t+1}^{e}\right)}{\sigma_{t}^{2}\left(m_{t+1}\right)}\right]\left[-\frac{\sigma_{t}^{2}\left(m_{t+1}\right)}{E_{t}\left(m_{t+1}\right)}\right]=\beta_{t,m,R^{e}}\lambda_{t,m}$$ (9)

where *λ*_*t*,*m*_ and **β**_**t**,*m*,**R**^**e**^_ can be interpreted as the price of risk and the risk loadings of the assets, respectively. Substituting Expression (7) into Expression (9):

$$E_{t}\left(R_{t+1}^{e}\right)=\beta_{t,f,R^{e}}\lambda_{t,f}$$ (10)

where **β**_**t**,**f**,**R**^**e**^_ is the *N*×*K* matrix of slopes of the time-series regressions of excess returns on the vector of factors **f**, and **λ**_**t,f**_ is the vector of slopes of the cross-sectional regression of expected returns on **β**_**t**,**f**,**R**^**e**^_.

When both the SDF and the excess returns are independent and identically distributed (i.i.d.) there is not difference between the conditional and unconditional versions of the model and we can remove *t* subscripts in the previous expressions. Specifically, in that case, the unconditional version of Expression (4), particularized to the linear SDF shown in Expression (7), can be written as follows:

$$E\left[\left(a+b^{\prime }f_{t+1}\right)R_{t+1}^{e}\right]=0_{N}$$ (11)

while Expression (10) results in:

$$E\left(R_{t+1}^{e}\right)=\beta_{f,R^{e}}\lambda_{f}$$ (12)

However, the pattern of predictability of asset returns and the time-varying nature of the equity risk premium make substantial differences emerge between conditional and unconditional asset pricing models [1, 49]. In this regard, it is important to note that a conditional asset pricing model does not necessarily imply an unconditional asset pricing model. In any case, according to [39], to determine the unconditional version of Expression (4) it is sufficient to consider that coefficients *a*_*t*_ and **b**_**t**_ in Expression (7) vary linearly with an instrument or vector of instruments observable at time *t*, where instruments should be forecasting variables for excess returns [2]. Remarkably [50], follow an analogous procedure to transform the conditional CAPM into an unconditional model. Consequently, in order to consider conditioning information into the model, we assume that the conditional nature of Expressions (4) and (7) results from the dependence of the coefficients *a*_*t*_ and **b**_**t**_ on an instrument *z* observable at time *t*. In this context, we can write the unconditional version of Expression (4) by parameterizing the coefficients *a*_*t*_ and **b**_**t**_ in Expression (7) as a function of *z*, as follows:

$$E\left\{\left[\left(a_{0}+a_{1}z_{t}\right)+{\left(b_{0}+b_{1}{\text{z}}_{\text{t}}\right)}^{\text{'}}f_{t+1}\right]R_{t+1}^{e}\right\}=0_{N}$$ (13)

Expression (13) shows that conditioning information adds two extra explanatory variables to the SDF in addition to the fundamental factors **f**_**t+1**_, namely, *z*_*t*_ and **f**_**t+1**_ *z*_*t*_. Following [2, 39], this allows us to write the following vector, which comprises the fundamental factors and the new variables that result from the information included in *z*_*t*_:

$$F_{t+1}={\left(\begin{array}{ccc} f_{t+1} & z_{t} & f_{t+1}z_{t} \end{array}\right)}^{'}$$ (14)

so that Expression (10) can be written as follows:

$$E\left(R_{t+1}^{e}\right)=\beta_{F,R^{e}}\lambda_{F}$$ (15)

In the next section we compare the performance of the unconditional and conditional versions of single-factor consumption models–i.e. the CCAPM and the CAPM–, as defined in Expressions (12) and (15), respectively, with that of the Fama-French three-factor model and the production models described below. For that purpose, we use the CCI as the instrument *z* in Expression (14).

Importantly, while the CAPM and the Fama-French three-factor model use traded factors as explanatory variables in the vector **f**, the CCAPM uses a non-traded factor–the per capita consumption growth–to explain expected returns. Consumption data is reported annually and quarterly, preventing Expressions (12) and (15) from being used at monthly or higher frequencies. However, it is always possible to determine the SDF implicit in Expressions (12) and (15) and subsequently estimate the factor-mimicking portfolio of the model as the projection of the SDF on the payoff space. Specifically, coefficients **λ**_**f**_ in Expression (12) allow us to determine the parameters *a* and **b** in Expression (11) straightforwardly, as follows [8] (pp. 106–107) (for Expression (15) this procedure is identical, simply by replacing **f** with **F**):

$$E\left[\left(1+b^{\prime }{\tilde{f}}_{t+1}\right)R_{t+1}^{e}\right]=0_{N}$$ (16)

$$b=-cov{\left(f_{t+1},f_{t+1}^{'}\right)}^{-1}\lambda_{f}$$ (17)

where ${\tilde{f}}_{t+1}$ is the vector of demeaned factors. Importantly, Expression (16) sets *a* = 1 to normalize the SDF to *E*_*t*_(*m*_*t*+1_) = 1. In this regard, it is worth noting that the pricing function does not identify the mean of the SDF when the payoff space comprises solely excess returns, so any arbitrary value for the coefficient *a* will produce exactly the same fitted values.

As noted above, the factor-mimicking portfolio of the model can be estimated as the projection of *m*_*t*+1_ –determined using Expressions (16) and (17)–on the payoff space, so that [8] (p. 67):

$$m_{t+1}=\text{proj}\left(m_{t+1}|\underline{R^{e}}\right)+\varepsilon_{t+1}$$ (18)

where proj(*m*_t+1_|*R*^*e*^) is the mimicking portfolio of the SDF (hereafter denoted as x*), *R*^*e*^ is the space of excess returns, and *ε*_*t*+1_ is the error term. Expression (18) is equivalent to the regression of *m*_*t*+1_ on the excess returns that constitute the test assets, that is:

$$m_{t+1}=w^{\prime }R_{t+1}^{e}+\varepsilon_{t+1}$$ (19)

where **w** is the vector of coefficients that result from the regression of *m*_*t*+1_ on $R_{t+1}^{e}$ or, equivalently, the vector of portfolio weights of the factor-mimicking portfolio x*, so that:

$$x_{t+1}^{*}=w^{\prime }R_{t+1}^{e}$$ (20)

Since the error term *ε*_*t*+1_ is orthogonal to $R_{t+1}^{e}$, the factor-mimicking portfolio carries exactly the same pricing information as *m*_*t*+1_. However, importantly, $x_{t+1}^{*}$ depends entirely on market data and can be determined at the same frequency as excess returns. Consequently, below we use Expression (20) to test the CCAPM on a monthly basis.

### Production models

While consumption models relate asset prices to the marginal utility that results from the investors’ first order condition, production models focus on the supply-side of the economy, exploiting the crucial role of investment and profitability of firms in equity returns. Specifically, production models assume that a firm chooses investments to maximize the cum-dividend value of equity, *V*_*t*_, which is given by the risk-adjusted present value of dividends, according to the following Bellman equation [17] (p. 209):

$$V_{t}=\underset{I_{t}}{\max}\left[D_{t}+E_{t}\left(m_{t+1}V_{t+1}\right)\right]$$ (21)

where *l*_*t*_ is the investment, and *D*_*t*_ is the dividend paid by the firm at time *t*. Most production models constitute special cases of Expression (21). In particular, the Fama-French five-factor model, as defined by [6], starts from a standard dividend discount model to write the inverse of the book-to-market equity ratio (hereafter, BE/ME ratio) as follows:

$$\frac{ME_{t}}{BE_{t}}=\frac{\sum_{\tau =1}^{\infty }\left(\prod_{t+\tau }-\Delta BE_{t+\tau }\right)/{\left(1+r\right)}^{\tau }}{BE_{t}}$$ (22)

where Π_*t*_ denotes the total equity earnings for period *t*, and *r* is the internal rate of return on expected dividends. Expression (22) suggests that a high BE/ME ratio (low ME/BE ratio) must either imply a low return on equity (ROE) or high returns over time and, consequently, ROE (i.e. profitability) should forecast future returns controlling for value. Additionally, controlling for *BE*_*t*_, *ME*_*t*_ and expected earnings, higher expected growth in book equity (i.e. investment) implies a lower expected return [6]. identify RMW and CMA as proxies for profitability and investment, respectively. These two factors combined with the Fama-French three-factor model result in the Fama-French five-factor model, which is represented in the form of an unconditional beta model, as defined in Expression (12), with the following vector of factors:

$$f={\left(\begin{array}{ccccc} \text{RMRF} & \text{SMB} & \text{HML} & \text{RMW} & \text{CMA} \end{array}\right)}^{\prime }$$ (23)

On the other hand, the *q*-model, as defined by [7], starts from Expression (21) to fully develop a production-based asset pricing model that assumes that the dividend at time *t* is determined as the difference between the gross profits paid to shareholders, Π_*t*_, and the cost of investment, Φ_*t*_. Thus, defining:

$$\prod_{t}=X_{t}K_{t}$$ (24)

$$\Phi_{t}=I_{t}+\frac{a}{2}{\left(\frac{I_{t}}{K_{t}}\right)}^{2}K_{t}$$ (25)

where *a* > 0 is a constant parameter, *K*_*t*_ is the productive capital, and the second term in the right-hand side of Expression (25) denotes the adjustment costs, then Expression (21) can be rewritten as follows, particularized to the case of two periods:

$$V_{t}=\underset{I_{t}}{\max}\left[X_{t}K_{t}-I_{t}-\frac{a}{2}{\left(\frac{I_{t}}{K_{t}}\right)}^{2}K_{t}+E_{t}\left(m_{t+1}X_{t+1}K_{t+1}\right)\right]$$ (26)

Expression (26) leads to the first principle of investment for firms, which says that:

$$1+a\frac{I_{t}}{K_{t}}=E_{t}\left(m_{t+1}X_{t+1}\right)$$ (27)

Combining Expressions (26) and (27) and operating the result yields the investment-CAPM, which can be written as follows [7, 43]:

$$r_{t+1}=\frac{X_{t+1}}{1+aI_{t}/K_{t}}$$ (28)

where *r*_*t*+1_ is the net stock return. The beta representation that results from Expression (28) is the *q*-model, which assumes the following vector of factors in Expression (12):

$$f={\left(\begin{array}{cccc} \text{RMRF} & \text{SMB} & r_{I/A} & r_{ROE} \end{array}\right)}^{\prime }$$ (29)

where *r*_*I*/*A*_ is the difference between the return on a portfolio of low investment stocks and the return on a portfolio of high investment stocks, and *r*_*ROE*_ is the difference between the return on a portfolio of high ROE stocks and the return on a portfolio of low ROE stocks.

Apart from some differences in the process followed to form the portfolios comprised in the factors, investment and profitability factors shown in Expressions (23) and (29) are very similar. In particular, both CMA and *r*_*I*/*A*_ use the growth of firms’ assets as sorting variable. Regarding profitability factors, RMW sorts stocks using revenues minus cost of goods sold, minus selling, general, and administrative expenses, minus interest expense, all divided by book equity. On the other hand, *r*_*ROE*_ uses ROE as sorting variable, which is essentially the same ratio as that used by RMW, except for the fact that ROE is determined after taxes.

In order to compare both models in a uniform basis and better analyze the apparent redundancy of HML with investment and profitability factors, in the next section we determine the three classic Fama-French factors following the standard methodology suggested by [14], using the approach followed by [7] to determine *r*_*I*/*A*_ and *r*_*ROE*_.

## Data and results

In this section we use Expression (12) to estimate all the unconditional models under consideration–namely, the CCAPM, the CAPM, the Fama-French three- and five-factor models, and the *q*-model–on the Tokyo Stock Exchange. Additionally, we use Expression (15) to estimate the conditional versions of the CCAPM and the CAPM, using the CCI as an instrument to capture the conditioning information. Since the performance of asset pricing models is sensitive to the periodicity of data [51], we study all models at three different frequencies. Specifically, we use an annual, quarterly and monthly periodicity. Given the annual and quarterly periodicity of consumption data, we use the factor-mimicking portfolio approach, as defined in Expressions (16), (17), (19) and (20), to study the performance of the CCAPM on a monthly basis. All these calculations allow us to analyze the informativeness of the CCI in asset pricing and to comprehensively compare the performance of consumption and production models on the Tokyo Stock Exchange.

### Data series

In order to test the models described in the previous section, we take the perspective of a Japanese representative investor, thus assuming not only the existence of a representative investor–i.e. perfect risk-sharing–, but also the national identity of this investor. Although most tests on the performance of asset pricing models in the US equity market adopt this perspective, research on international equity markets often takes the perspective of a US investor investing in a foreign market. For example [52], conduct a research on the performance of the Fama-French five-factor model on different countries and geographical areas, measuring all returns in dollars and using the one-month US Treasury bill rate as the risk-free rate. Conversely, we measure returns in local currency, using the three-month Treasury Bill rate for Japan as the risk-free rate. This helps minimize currency effects on asset returns, allowing us to focus exclusively on the dynamics of equity risk premiums from a domestic perspective.

The previous assumption largely conditions the source of data used in the paper for stock returns. Specifically, while many studies on the cross-sectional properties of stock returns use the data made publicly available by Kenneth French on his website, the Japanese representative investor assumption prevents us from using those series, as these data are provided in dollars. In order to avoid errors arising from changing data to the local currency, we compile all stock data from the Datastream database.

We test all models on different sets of portfolios, which comprise all stocks listed in the Tokyo Stock Exchange, for the period from July 1992 to June 2018 (all data is publicly available at [53]). For that purpose, we collect data for the following series, on a monthly basis: (i) total return index (RI series), which allows us to determine asset returns, (ii) market value (MV series), (iii) market-to-book equity (PTBV series), (iv) total assets (WC02999 series), (v) return on equity (WC08301 series), (vi) price-to-cash flow ratio (PC series), and (vii) dividend yield (DY series). We exclude non-common equity securities from Datastream data following the generic rules suggested by [54]. We also exclude stocks with less than twelve observations in the period from July 1992 to June 2018. Accordingly, our sample comprises a total number of 5,312 stocks. As noted, we use the three-month Japanese Treasury Bill rate, as provided by the OECD database, as a proxy for the risk-free rate.

We follow the methodology suggested by [14] to create three sets of portfolios, which constitute our test assets. Specifically: (i) 25 size-BE/ME portfolios, (ii) 20 momentum portfolios, and (iii) 25 price-to-cash flow-dividend yield portfolios (hereafter, P/CF-DY portfolios). Additionally, we follow [14] and [7] to determine market factors, namely, RMRF, SMB, HML, *r*_*I*/*A*_ and *r*_*ROE*_. Importantly [55], attribute the strong explanatory power of different asset pricing models studied in the literature to the specific test assets typically used in empirical research and, in particular, to the strong factor structure of size-BE/ME portfolios. To avoid this drawback, the authors suggest using other portfolios to test models, in addition to size-BE/ME portfolios. We have followed this recommendation to configure our test assets.

We compile all macroeconomic data series from the OECD Statistics section. Regarding consumption data, although the CCAPM establishes the consumption growth in nondurable goods and services as the only explanatory variable for asset prices, we use total consumption data instead (‘Private final consumption expenditure’ series, CQR measure), following [56], which show that total consumption of households fits the cross-section of expected returns better than consumption in nondurables and services. We use the population series for Japan, as provided by the OECD, to determine per capita consumption growth.

We collect the CCI data series for Japan, for the period from July 1991 to June 2018, on a monthly basis, from the OECD Statistics section (dataset ‘Composite Leading Indicators’, MEI). This indicator comprises the following data series: (i) the inverse of the ratio of inventories to shipments, (ii) the quotient of the volume of imports and exports, (iii) the inverse of the loans to deposits ratio, (iv) the number of hours worked in the manufacturing industry, (v) the work started for dwellings, (vi) the share prices in the TOPIX index, (vii) the spread of interest rates, and (viii) the Small Business Survey on sales trend. Our data show that the annual CCI is positively correlated with the gross domestic product growth rate (Δ*GDP*), while it is poorly correlated with the consumer price index (CPI), with correlations of 0.46 and -0.05, respectively. Following [57], we perform all calculations below using the demeaned CCI.

Table 1 shows the main summary statistics for portfolios and factors under consideration. Panel A shows that the size effect–that is, the fact that stocks with lower market equity provide higher returns, and vice versa–works as expected, with portfolios comprising the smallest stocks providing significantly higher returns. However, the value effect–that is, the fact that stocks with higher BE/ME provide higher returns, and vice versa–exhibits a less clear pattern, which is consistent with the sharp reduction in the value premium experienced in other markets, such as the New York Stock Exchange [8] (p. 452).

**Table 1:** Summary statistics.

Panel A: 25 portfolios size-BE/ME											
	BE/ME quintiles						BE/ME quintiles				
Size	Low	2	3	4	High	Size	Low	2	3	4	High
	Means						St. Dev.				
Small	5,17	4,76	3,85	3,09	3,12	Small	16,71	13,84	10,31	7,90	7,97
2	2,77	2,64	2,18	2,09	2,38	2	11,24	8,67	7,02	6,74	7,28
3	2,31	1,96	1,79	1,66	2,15	3	9,68	7,46	6,48	6,32	7,14
4	1,97	1,24	1,47	1,78	2,12	4	8,10	5,86	5,93	6,30	7,56
Big	0,84	1,06	1,53	1,45	1,72	Big	6,37	5,65	6,27	6,95	9,44
Panel B: 20 portfolios momentum											
	Means						St. Dev.				
Low-5	2,23	1,47	1,18	1,28	1,26	Low-5	10,25	9,31	7,33	7,02	7,25
6–10	0,84	0,90	1,17	1,98	1,03	6–10	6,77	6,73	6,36	6,22	6,48
11–15	1,08	0,89	1,18	1,02	1,00	11–15	6,15	5,59	6,24	5,76	5,80
15-High	0,88	0,87	1,28	1,24	2,08	15-High	5,89	5,82	6,54	6,84	9,25
Panel C: 25 portfolios P/CF-DY											
	DY quintiles						DY quintiles				
P/CF	Low	2	3	4	High	P/CF	Low	2	3	4	High
	Means						St. Dev.				
Low	2,70	1,07	1,46	0,97	1,80	Low	11,36	10,33	8,65	7,64	9,21
2	1,90	0,84	1,39	1,71	1,47	2	9,36	6,64	6,98	6,61	7,63
3	1,26	1,13	1,17	1,41	1,36	3	8,05	6,11	6,27	6,13	6,01
4	1,44	0,89	1,06	1,18	1,01	4	8,36	5,93	5,37	6,43	6,52
High	1,41	0,89	0,72	1,17	0,75	High	9,12	6,28	5,76	8,09	6,05
Panel D: Market factors and macroeconomic series											
	RMRF	SMB	HML	RMW	CMA		Δ*C* (quarters)		Δ*C* (years)		CCI
Means	1,17	1,22	0,14	0,84	-0,10	Means	0,15	Means	0,44	Means	0,00
St. Dev.	5,72	4,60	3,39	3,07	2,96	St. Dev.	2,38	St. Dev.	1,20	St. Dev.	124,15

Note: We compile monthly series for all stocks listed in the Tokyo Stock Exchange from the Datastream database, for the period from July 1992 to June 2018. Using this data, we form the following portfolios: (i) 25 portfolios size-BE/ME, (ii) 20 momentum portfolios, and (iii) 25 portfolios P/CF-DY. In order to determine excess returns, when appropriate we use the three-month interest rate of the Treasury Bill for Japan. Panels A and C use rows and columns to represent the quintiles for the first and second sorting variable, respectively. Panel C represents momentum demi-deciles in rows. All results are determined using monthly data, unless otherwise indicated. Means and standard deviations are percentages.

On the other hand, Panel B in Table 1 suggests a U-shape curve in the mean returns of momentum portfolios, with portfolios comprising stocks with the highest and lowest past one-year return providing the highest returns. Regarding P/CF-DY portfolios, Panel C in Table 1 shows that, in general, the higher the price-to-cash flow ratio, the lower the mean return, and vice versa, which is consistent with the results provided by most ratios that scale prices by some firm-specific characteristic. Conversely, the dividend yield does not deliver a clear pattern in average returns.

Table 1, Panel D shows means and standard deviations of factors used as explanatory variables. As shown, the mean of RMRF is relatively high (1.17% on a monthly basis) compared to the result achieved by [52], which provide a near-zero mean return for the period from 1990 to 2015. However, the fact that the authors measure all returns in dollars and use the US Treasury bill rate as a proxy for the risk-free rate makes these results not directly comparable. Regarding macroeconomic series, Panel D in Table 1 shows that, for the period under study, Japan exhibits an average consumption growth of 0.44% using annual consumption data, which is a relatively low rate compared to other countries. Additionally, Panel D shows that the CCI is highly volatile, with a standard deviation of 124.15%, on a monthly basis.

Table 2 summarizes the correlations between our test assets and factors. As shown, most portfolios exhibit high positive correlations with RMRF and RMW, while size-BE/ME portfolios are also significantly correlated with SMB. Remarkably, all portfolios under consideration exhibit a weak negative correlation with quarterly consumption growth. However, it is important to mention that these correlations do not condition the performance of asset pricing models. Instead, according to Expression (8), it is the correlation of expected excess returns with the covariances between excess returns and factors that determines the size of pricing errors.

**Table 2:** Correlations.

Panel A: 25 portfolios size-BE/ME											
	BE/ME quintiles						BE/ME quintiles				
Size	Low	2	3	4	High	Size	Low	2	3	4	High
	RMRF						SMB				
Small	0,53	0,40	0,47	0,60	0,60	Small	0,54	0,43	0,55	0,50	0,49
2	0,53	0,65	0,69	0,70	0,65	2	0,51	0,52	0,53	0,51	0,46
3	0,66	0,69	0,78	0,79	0,76	3	0,52	0,40	0,37	0,36	0,37
4	0,79	0,84	0,83	0,80	0,79	4	0,32	0,27	0,30	0,25	0,15
Big	0,94	0,95	0,87	0,77	0,68	Big	-0,11	-0,15	-0,03	0,05	0,02
	HML						RMW				
Small	-0,31	-0,16	-0,13	0,02	0,05	Small	0,49	0,30	0,45	0,43	0,38
2	-0,16	-0,19	-0,03	-0,01	0,18	2	0,54	0,53	0,49	0,47	0,42
3	-0,16	-0,12	-0,02	0,09	0,13	3	0,53	0,56	0,51	0,47	0,42
4	-0,20	-0,03	-0,02	0,10	0,14	4	0,55	0,56	0,49	0,52	0,34
Big	-0,20	-0,09	0,03	0,16	0,18	Big	0,44	0,48	0,53	0,45	0,43
	CMA						Δ*C* (quarters)				
Small	0,07	0,08	0,11	0,17	0,23	Small	-0,29	-0,14	-0,26	-0,32	-0,37
2	0,04	0,11	0,15	0,20	0,30	2	-0,30	-0,24	-0,36	-0,33	-0,31
3	0,08	0,20	0,17	0,24	0,28	3	-0,30	-0,14	-0,23	-0,31	-0,33
4	0,03	0,14	0,17	0,23	0,26	4	-0,18	-0,21	-0,22	-0,30	-0,26
Big	0,08	0,17	0,22	0,25	0,26	Big	-0,05	-0,12	-0,18	-0,29	-0,22
Panel B: 20 portfolios momentum											
	RMRF						SMB				
Low-5	0,70	0,74	0,78	0,81	0,78	Low-5	0,18	0,06	0,09	0,04	0,00
6–10	0,80	0,79	0,85	0,86	0,84	6–10	0,04	0,01	0,01	-0,01	-0,07
11–15	0,83	0,84	0,83	0,85	0,87	11–15	-0,01	0,01	-0,05	0,04	0,02
15-High	0,88	0,87	0,86	0,84	0,78	15-High	-0,06	0,02	-0,05	0,03	0,07
	HML						RMW				
Low-5	0,01	0,04	-0,08	-0,06	0,01	Low-5	0,49	0,46	0,46	0,46	0,43
6–10	-0,05	0,00	-0,03	-0,10	-0,02	6–10	0,46	0,45	0,48	0,42	0,31
11–15	-0,01	-0,02	-0,03	-0,04	0,00	11–15	0,49	0,44	0,39	0,46	0,50
15-High	-0,11	-0,09	-0,13	-0,12	-0,22	15-High	0,39	0,36	0,41	0,46	0,46
	CMA						Δ*C* (quarters)				
Low-5	0,26	0,24	0,23	0,24	0,27	Low-5	-0,22	-0,20	-0,22	-0,23	-0,18
6–10	0,19	0,22	0,22	0,19	0,17	6–10	-0,25	-0,25	-0,17	-0,13	-0,09
11–15	0,22	0,26	0,14	0,22	0,20	11–15	-0,18	-0,17	-0,12	-0,10	-0,09
15-High	0,17	0,09	0,01	0,02	-0,05	15-High	-0,02	-0,09	-0,04	-0,01	-0,09
Panel C: 25 portfolios P/CF-DY											
	DY quintiles						DY quintiles				
P/CF	Low	2	3	4	High	P/CF	Low	2	3	4	High
	RMRF						SMB				
Low	0,74	0,76	0,82	0,75	0,62	Low	0,28	-0,04	-0,02	0,06	0,09
2	0,76	0,83	0,75	0,74	0,71	2	0,16	-0,09	-0,03	0,05	0,04
3	0,69	0,88	0,82	0,81	0,76	3	0,19	-0,15	-0,02	0,14	0,18
4	0,80	0,90	0,85	0,77	0,73	4	0,18	-0,10	0,03	0,05	0,20
High	0,72	0,91	0,82	0,75	0,74	High	0,26	-0,11	0,01	-0,05	0,17
	HML						RMW				
Low	0,04	-0,10	0,03	0,02	0,21	Low	0,61	0,47	0,39	0,46	0,45
2	-0,05	-0,13	-0,04	0,11	0,08	2	0,55	0,41	0,55	0,50	0,53
3	-0,05	-0,12	0,05	0,03	0,13	3	0,45	0,41	0,51	0,46	0,41
4	-0,05	-0,19	-0,03	0,01	0,10	4	0,45	0,37	0,48	0,46	0,40
High	-0,19	-0,25	-0,04	-0,01	0,03	High	0,51	0,39	0,49	0,12	0,47
	CMA						Δ*C* (quarters)				
Low	0,22	0,09	0,20	0,23	0,25	Low	-0,27	-0,11	-0,10	-0,27	-0,18
2	0,17	0,10	0,20	0,22	0,20	2	-0,23	-0,09	-0,12	-0,17	-0,19
3	0,14	0,10	0,22	0,22	0,21	3	-0,24	-0,13	-0,16	-0,21	-0,27
4	0,20	0,10	0,19	0,26	0,27	4	-0,19	-0,09	-0,15	-0,18	-0,26
High	0,08	0,01	0,23	0,19	0,27	High	-0,12	-0,03	-0,14	-0,13	-0,31

Note: See notes to Table 1. Results are in decimals.

### Predictive power of consumer sentiment

Any instrument useful for capturing the conditioning information used by investors must have some predictive power to forecast asset returns or macroeconomic indicators [8] (p. 135). Accordingly, Table 3 summarizes the results of different forecasting regressions, using the CCI for Japan as a predictor. Panel A shows the regression results of the AR(1) model for the CCI at different horizons. As shown, consumer sentiment is strongly persistent in the short-run, providing a slope coefficient and a *R*^2^ statistic of 0.895 and 0.801, respectively, for the three-month horizon. However, that persistence declines rapidly over time, providing negligible estimates for two- and five-year horizons. These results contrast with those provided by other predictors, such as the dividend yield or the consumption-wealth ratio, which deliver one-year slope coefficients of around 0.9 and 0.6, respectively [1].

**Table 3:** Predictive power analysis for the CCI.

Panel A: CCI forecasting regressions								
*CCI*_*t*_ = *a* + *bCCI*_*t–k*_ + *ε*_*t*_								
	3 months		1 year		2 years		5 years	
Slope	,895		,364		-,272		,030	
	(16,600)		(2,197)		(-1,453)		(,277)	
*R*^2^	,801		,137		,086		,001	
Panel B: Return forecasting regressions using the CCI as a regressor								
*RMRF*_*t*_ = *a* + *bCCI*_*t–k*_ + *ε*_*t*_								
	3 months		1 year		2 years		5 years	
Slope	,011		-,010		-,096		-,279	
	(,957)		(-,314)		(-1,575)		(-3,701)	
*R*^2^	,012		,002		,069		,203	
Panel C: Return forecasting regressions using the GDP as a regressor								
*RMRF*_*t*_ = *a* + *b*Δ*GDP*_*t–k*_ + *ε*_*t*_								
	3 months		1 year		2 years		5 years	
Slope	-,104		-4,125		-5,487		-7,798	
	(-,436)		(-1,469)		(-1,201)		(-1,288)	
*R*^2^	,002		,071		,052		,033	
Panel D: Return forecasting regressions using the CCI and the GDP as regressors								
*RMRF*_*t*_ = *a* + *bCCI*_*t–k*_ + *c*Δ*GDP*_*t–k*_ + *ε*_*t*_								
	3 months		1 year		2 years		5 years	
	*CCI*	Δ*GDP*	*CCI*	Δ*GDP*	*CCI*	Δ*GDP*	*CCI*	Δ*GDP*
Slope	,011	-,118	,031	-5,174	-,072	-3,054	-,299	2,668
	(1,005)	(-,496)	(,841)	(-1,504)	(-,955)	(-,582)	(-3,289)	(,315)
*R*^2^	,015		,083		,081		,206	

Notes: We compile monthly series for all stocks listed in the Tokyo Stock Exchange from the Datastream database, for the period from July 1992 to June 2018, and determine RMRF accordingly. We compile monthly series for the CCI and the GDP for Japan, as provided by the OECD. Panel A shows the slopes and the *R*^2^ statistics for the AR(1) process of the CCI. Panels B, C and D show the slopes and *R*^2^ statistics for the regressions of RMRF on the following lagged variables, respectively: (i) the CCI, (ii) Δ*GDP*, and (iii) the CCI and Δ*GDP*. The *t*-statistics are in parentheses. We correct standard errors for the autocorrelation that results from overlapping returns, following the [58] methodology.

Panel B in Table 3 shows the results of the forecast regression analysis for the value-weighted market portfolio, RMRF, using the CCI as a predictor. Additionally, in order to study the potential shifts in the predictive power of the CCI in the presence of other macroeconomic regressors, Panel C shows the regression results using Δ*GDP* as a forecasting variable, while Panel D uses both the CCI and Δ*GDP* as predictors. As shown in Panel B, although three-month and one-year estimates are virtually negligible, the predictive power of the CCI increases as the time interval expands, which is a common feature of most asset return predictors. Specifically, although the one-year slope coefficient amounts to -0.01, it reaches -0.279 for a five-year horizon. Similarly, the *R*^2^ statistic increases from 0.002 to 0.203, for those time intervals. Remarkably, the five-year slope coefficient is statistically significant, with standard errors corrected for the autocorrelation that results from overlapping returns, according to the [43] methodology.All slope coefficients shown in Table 3, Panel B, are negative with the sole exception of the three-month estimate. This is consistent with the cyclical nature of consumer sentiment, which means that the higher the CCI, the higher the market prices and, consequently, the lower the expected returns. Regarding three-month estimates, the positive slope coefficient suggests a continuation of the recent trend in stock returns, which is consistent with the momentum anomaly, as described by [14] and [42].

Panels C and D in Table 3 show that the same conclusions hold when we include Δ*GDP* as a predictor. Indeed, Panel C shows that, although the GDP exhibits some predictive power for the one-year forecast, in the long-run its importance declines sharply, providing a *R*^2^ statistic of 0.033 for the five-year forecast. Consistently, Panel D in Table 3 shows that most of the estimates for the CCI, as well as the *R*^2^ statistics, remain almost unchanged from those shown in Panel B, which is indicative of the small predictive power of Δ*GDP*.

In summary, although our results suggest that the predictive power of the CCI for Japan is lower than that of other predictors widely studied in the literature, the fact that consumer sentiment provides significantly better estimates as the time interval expands, together with the high volatility of the CCI, makes us expect that consumer sentiment can be a good instrument to account for conditioning information in cross-sectional consumption models.

### Model results

In order to estimate the coefficients of the asset pricing models described above, we use a two-pass cross-sectional regression (CSR) methodology, following a procedure similar to that pioneered by [59] and [60]. According to this methodology, in a first stage, the time-series regressions of excess returns on factors provide the beta coefficients of the model (i.e. Expressions (12) and (15), depending on the unconditional or conditional nature of the model, respectively). In a second stage, the cross-sectional regression of average excess returns on betas produces lambda coefficients. Although this procedure does not guarantee that the model correctly prices the factors–as is the case in a purely time-series approach, where the factors are in the payoff space and *λ* = *E*(*f*) is assumed–, it constitutes a well-established methodology in the asset pricing literature, which allows the model to fit expected payoffs to betas and determine the prices of risk even when the factors are not traded.

Nevertheless, the two-pass CSR methodology involves some specific issues that can lead to misleading estimates. In particular, the fact that errors are typically cross-sectionally correlated at a given time, the estimated nature of betas, and the problems arising from measurement errors can severely distort model estimates [8] (pp. 247–248). Thus, while the cross-sectional correlation can result in misleading standard errors, the fact that betas are generated regressors not only can lead to biased *t*-statistics, but can also result in inconsistent estimators. In this regard [61, 62], examine a wide range of implications of the use of constructed variables on the properties of estimators, including their consistency, efficiency and potential to provide valid inferences, while [63] study the effects of estimated regressors in models with risk terms. Notably, the classic procedure developed by [60] (hereafter, the Fama-MacBeth procedure) helps to flexibly correct standard errors for cross-sectionally correlated errors, while other approaches, such as that suggested by [64], allow correcting standard errors for the fact that betas are estimated.

Importantly [8], (pp. 247–251) explains that, under certain conditions, the Fama-MacBeth procedure is numerically equivalent to a pooled regression, and emphasizes the benefits of mapping this approach into the generalized method of moments (GMM), developed by [65]. Notably [66], introduces a GMM estimator that relies on distance instruments to estimate panel data regression models containing errors in variables [32, 33]. use this approach to test the Fama-French five-factor model augmented with illiquidity measures, concluding that, in general, the most significant factor is RMRF, with measurement errors largely determining this result. On the other hand [34], use a distance instrument-based algorithm into a GMM framework, to study the impact of illiquidity on the returns of twelve sector portfolios. Consistently with [32, 33], the authors conclude that the only relevant factor according to their dynamic GMM approach is the market risk premium.

Given the aforementioned issues related to the two-pass CSR methodology, in order to allow comparison between our results and previous research that analyzes the effects of conditioning variables, we estimate the models described above by mapping OLS regressions into GMM, following [8] (pp. 241–242). This allows us to simultaneously estimate betas and lambdas in Expressions (12) and (15) and correct standard errors for cross-sectional autocorrelation in a manner similar to the Fama-MacBeth procedure, but additionally correcting for the fact that betas are generated regressors. Specifically, following [8] (p. 241), we define the following vector of moments:

$$g_{T}\left(b\right)=\left\{E\begin{array}{c} E\left(R_{t}^{e}-a-\beta X_{t}\right)\\ \left[\left(R_{t}^{e}-a-\beta X_{t}\right)X_{t}\right]\\ E\left(R_{t}^{e}-\beta \right) \end{array}\right\}$$ (30)

where **a** is the vector of intercepts of the time-series regressions, and **X**_t_ is the vector of factors, that is, **f**_**t**_ or **F**_**t**_, depending on the unconditional or conditional nature of the model, respectively. In order to allow GMM to reproduce the two-pass CSR estimates, we weight the moments in Expression (30) using the following matrix [8] (p. 242) (**I** denotes the identity matrix):

$$a_{T}=\left(\begin{array}{cc} I_{2N} & \\ & \beta^{\prime } \end{array}\right)$$ (31)

so that:

$$a_{T}g_{T}\left(\hat{b}\right)=0_{3N}$$ (32)

In order to estimate standard errors and the distribution of moments, we use the standard GMM formulation (see [8] (pp. 203–204)) and a spectral density matrix **S** with zero leads and lags, as follows:

$$S=E\left\{\left[\begin{array}{c} R_{t}^{e}-a-\beta X_{t}\\ \left(R_{t}^{e}-a-\beta X_{t}\right)X_{t}\\ R_{t}^{e}-\beta \end{array}\right]{\left[\begin{array}{c} R_{t}^{e}-a-\beta X_{t}\\ \left(R_{t}^{e}-a-\beta X_{t}\right)X_{t}\\ R_{t}^{e}-\beta \end{array}\right]}^{'}\right\}$$ (33)

Tables 4–6 show the regression results provided by all models under consideration, namely, the CCAPM and the CAPM, both conditional on consumer sentiment and unconditional, the Fama-French three- and five-factor models, and the *q*-model. In order to accurately evaluate the performance of these models, we study the anomaly portfolios described above separately. In particular, Table 4 shows the regression results achieved for size-BE/ME portfolios, while Tables 5 and 6 do the same for momentum portfolios and P/CF-DY portfolios. Each table comprises three panels, the first showing annual results and the second and third showing quarterly and monthly results, respectively.

**Table 4:** Regression results for 25 portfolios size-BE/ME.

			CCAPM	Market factor models					Instrument				MAE	
Row	Model	Intercept	*λ*_Δ*C*_	*λ*_*RMRF*_	*λ*_*SMB*_	*λ*_*HML*_	*λ*_*I/A*_	*λ*_*ROE*_	*λ*_*CCI*_	*λ*_CCI·ΔC_	*λ*_*CCI·RMRF*_	*R*^2^	(%)	*J*-test
Panel A: Annual data														
1	Unconditional CCAPM	,010	,032									,500	9,29	87,878
		(,051)	(1,157)									,363		(,000)
2	Conditional CCAPM	-,075	,019						-2,005	,007		,948	3,76	101,253
		(-,403)	(,845)						(-1,723)	(,313)		,462		(,000)
3	Unconditional CAPM	,051		,276								,542	10,71	327,267
		(,692)		(2,387)								,359		(,000)
4	Conditional CAPM	-,020		,209					-1,490		,039	,944	4,33	163,805
		(-,179)		(1,772)					(-1,392)		(,232)	,611		(,000)
5	Fama-French (3 factors)	,063		,134	,193	-,020						,935	3,97	347,288
		(,755)		(1,628)	(2,634)	(-,194)						,900		(,000)
6	Fama-French (5 factors)	-,022		,173	,223	-,010	,062	,108				,971	2,84	141,455
		(-,449)		(2,031)	(3,120)	(-,099)	(,965)	(1,711)				,926		(,000)
7	*q*-model	-,055		,196	,242		,095	,097				,963	3,23	163,576
		(-,976)		(2,222)	(3,302)		(1,592)	(1,477)				,926		(,000)
Panel B: Quarterly data														
8	Unconditional CCAPM	,009	-,035									,673	1,42	19,257
		(,461)	(-3,126)									,549		(,686)
9	Conditional CCAPM	,006	,002						-1,876	,017		,892	,89	11,150
		(,265)	(,091)						(-1,967)	(,595)		,785		(,960)
10	Unconditional CAPM	-,056		,124								,482	1,81	30,274
		(-1,784)		(3,248)								,324		(,142)
11	Conditional CAPM	,021		,007					-2,523		-,055	,892	,88	9,613
		(,342)		(,110)					(-2,444)		(-,400)	,519		(,984)
12	Fama-French (3 factors)	,034		-,002	,056	-,013						,846	1,06	35,813
		(,938)		(-,065)	(5,088)	(-,785)						,762		(,023)
13	Fama-French (5 factors)	,030		,005	,050	-,013	-,004	,022				,860	1,02	29,666
		(,882)		(,154)	(4,378)	(-,747)	(-,140)	(,998)				,673		(,056)
14	*q*-model	-,005		,035	,056		,002	,010				,831	1,09	31,487
		(-,211)		(1,411)	(4,816)		(,105)	(,392)				,723		(,049)
Panel C: Monthly data														
15	Unconditional CCAPM	,005	-,225									,756	,37	56,382
		(1,476)	(-5,495)									,674		(,000)
16	Conditional CCAPM	,011	-,371									,765	,36	37,984
		(2,828)	(-5,222)									,601		(,026)
17	Unconditional CAPM	-,002		,026								,145	,75	70,804
		(-,311)		(2,821)								,072		(,000)
18	Conditional CAPM	,017		-,007					-2,171		-,073	,860	,30	10,985
		(1,106)		(-,414)					(-2,434)		(-1,483)	,555		(,963)
19	Fama-French (3 factors)	,002		,008	,017	,001						,790	,37	47,691
		(,269)		(,853)	(5,897)	(,225)						,743		(,001)
20	Fama-French (5 factors)	,008		,002	,017	-,004	,001	,021				,857	,31	28,410
		(,856)		(,182)	(5,451)	(-,995)	(,224)	(2,841)				,769		(,076)
21	*q*-model	-,002		,011	,019		,003	,010				,819	,32	40,127
		(-,301)		(1,269)	(6,080)		(,628)	(1,812)				,743		(,005)

Notes: We compile monthly series for all stocks listed in the Tokyo Stock Exchange from the Datastream database, for the period from July 1992 to June 2018. Using this data, we form 25 size-BE/ME portfolios. To determine excess returns, we use the three-month Treasury Bill rate for Japan. Depending on the model, we use the consumption growth, the market portfolio or the Fama-French factors-*q* factors as explanatory variables. In models 2, 4, 9, 11, 16 and 18, we scale factors using the CCI as an instrument. We map the two-pass CSR procedure into GMM to estimate all models, assuming a spectral density matrix with zero leads and lags. We use the same spectral density matrix to run the *J*-test. The table displays two rows for each model, where the first row shows the coefficient estimates and the second row the *t*-statistics. For each model, the columns labeled ‘*R*^2^’ shows the adjusted OLS and GLS *R*^2^ statistics, in that order. All *p*-values resulting from the *J*-tests are in parentheses. Coefficients shown in Panel C for the CCAPM are determined using the factor-mimicking portfolio of the model, as defined in Expression (20), in order to transform the coefficients that result from quarterly consumption data into monthly estimates.

**Table 5:** Regression results for 20 momentum portfolios.

			CCAPM	Market factor models					Instrument				MAE	
Row	Model	Intercept	*Λ*_ΔC_	*λ*_*RMRF*_	*λ*_*SMB*_	*λ*_*HML*_	*λ*_*I/A*_	*λ*_*ROE*_	*λ*_*CCI*_	*λ*_CCI·ΔC_	*λ*_*CCI·RMRF*_	*R*^2^	(%)	*J*-test
Panel A: Annual data														
1	Unconditional CCAPM	-,060	,027									,657	3,52	25,209
		(-,355)	(,926)									-,076		(,119)
2	Conditional CCAPM	,042	,006						-1,291	,001		,868	1,93	47,836
		(,367)	(,323)						(-1,663)	(,041)		,533		(,000)
3	Unconditional CAPM	,067		,126								,761	2,49	92,418
		(1,400)		(1,566)								,747		(,000)
4	Conditional CAPM	,069		,113					-,971		-,156	,935	1,47	61,310
		(1,041)		(1,201)					(-1,250)		(-1,373)	,864		(,000)
5	Fama-French (3 factors)	,039		,142	,040	,064						,930	1,67	69,934
		(,769)		(1,724)	(,457)	(,577)						,782		(,000)
6	Fama-French (5 factors)	,084		,110	-,047	,027	,098	-,002				,941	1,40	59,946
		(1,364)		(1,275)	(-,439)	(,330)	(1,931)	(-,058)				,903		(,000)
7	*q*-model	,050		,128	,014		,134	,026				,921	1,60	65,926
		(,773)		(1,479)	(,127)		(2,528)	(,587)				,756		(,000)
Panel B: Quarterly data														
8	Unconditional CCAPM	,028	-,011									,192	,75	23,860
		(2,379)	(-1,272)									,115		(,160)
9	Conditional CCAPM	,023	,003						-1,190	,005		,755	,42	9,242
		(1,396)	(,211)						(-2,098)	(,228)		,654		(,903)
10	Unconditional CAPM	-,031		,070								,841	,41	14,714
		(-1,538)		(3,047)								,801		(,682)
11	Conditional CAPM	-,007		,046					-,364		-,039	,834	,39	12,832
		(-,272)		(1,666)					(-,418)		(-1,062)	,818		(,685)
12	Fama-French (3 factors)	-,031		,069	,012	-,002						,840	,40	14,141
		(-1,660)		(3,155)	(,573)	(-,102)						,775		(,588)
13	Fama-French (5 factors)	-,028		,067	-,004	-,011	,042	,010				,857	,35	10,805
		(-1,398)		(2,980)	(-,164)	(-,662)	(2,976)	(,624)				,824		(,701)
14	*q*-model	-,030		,069	-,005		,040	,008				,863	,35	11,011
		(-1,467)		(3,017)	(-,206)		(3,052)	(,490)				,840		(,752)
Panel C: Monthly data														
15	Unconditional CCAPM	,010	-,015									,168	,24	30,187
		(3,143)	(-1,254)									,090		(,036)
16	Conditional CCAPM	,008	-,081									,602	,18	22,911
		(2,565)	(-3,737)									,574		(,194)
17	Unconditional CAPM	-,015		,028								,830	,12	12,741
		(-2,252)		(3,521)								,815		(,807)
18	Conditional CAPM	-,012		,025					-,096		-,010	,826	,12	12,806
		(-1,156)		(2,227)					(-,219)		(-,590)	,809		(,687)
19	Fama-French (3 factors)	-,010		,021	,007	-,004						,842	,12	12,110
		(-1,510)		(2,701)	(,999)	(-,462)						,804		(,736)
20	Fama-French (5 factors)	-,009		,020	,006	-,003	,009	,000				,828	,11	11,801
		(-1,194)		(2,361)	(,745)	(-,453)	(2,065)	(-,060)				,793		(,622)
21	*q*-model	-,009		,020	,006		,009	,000				,839	,11	11,835
		(-1,204)		(2,387)	(,766)		(2,127)	(-,060)				,810		(,691)

Notes: We compile monthly series for all stocks listed in the Tokyo Stock Exchange from the Datastream database, for the period from July 1992 to June 2018. Using this data, we form 20 momentum portfolios. To determine excess returns, we use the three-month Treasury Bill rate for Japan. Depending on the model, we use the consumption growth, the market portfolio or the Fama-French factors-*q* factors as explanatory variables. In models 2, 4, 9, 11, 16 and 18, we scale factors using the CCI as an instrument. We map the two-pass CSR procedure into GMM to estimate all models, assuming a spectral density matrix with zero leads and lags. We use the same spectral density matrix to run the *J*-test. The table displays two rows for each model, where the first row shows the coefficient estimates and the second row the *t*-statistics. For each model, the columns labeled ‘*R*^2^’ shows the adjusted OLS and GLS *R*^2^ statistics, in that order. All *p*-values resulting from the *J*-tests are in parentheses. Coefficients shown in Panel C for the CCAPM are determined using the factor-mimicking portfolio of the model, as defined in Expression (20), in order to transform the coefficients that result from quarterly consumption data into monthly estimates.

**Table 6:** Regression results for 25 portfolios P/CF-DY.

			CCAPM	Market factor models					Instrument				MAE	
Row	Model	Intercept	*Λ*_ΔC_	*λ*_*RMRF*_	*λ*_*SMB*_	*λ*_*HML*_	*λ*_*I/A*_	*λ*_*ROE*_	*λ*_*CCI*_	*λ*_CCI·ΔC_	*λ*_*CCI·RMRF*_	*R*^2^	(%)	*J*-test
Panel A: Annual data														
1	Unconditional CCAPM	,017	,019									,633	3,26	137,980
		(,261)	(1,178)									-,393		(,000)
2	Conditional CCAPM	,020	,012						-1,055	,009		,719	2,97	163,202
		(,274)	(1,599)						(-1,628)	(,592)		-1,508		(,000)
3	Unconditional CAPM	,092		,103								,526	4,17	330,285
		(2,425)		(1,334)								,501		(,000)
4	Conditional CAPM	,006		,160					-1,044		,006	,734	2,95	164,326
		(,091)		(1,657)					(-1,724)		(,052)	,228		(,000)
5	Fama-French (3 factors)	,055		,106	,124	,029						,826	2,66	272,958
		(1,564)		(1,373)	(1,571)	(,491)						,501		(,000)
6	Fama-French (5 factors)	,056		,104	,099	,016	,095	,036				,849	2,24	258,905
		(1,807)		(1,352)	(1,332)	(,276)	(2,044)	(,977)				,518		(,000)
7	*q*-model	,046		,112	,117		,099	,048				,845	2,25	243,635
		(1,250)		(1,425)	(1,430)		(2,095)	(1,214)				,580		(,000)
Panel B: Quarterly data														
8	Unconditional CCAPM	,021	-,018									,516	,73	27,216
		(1,746)	(-2,345)									,249		(,247)
9	Conditional CCAPM	,018	-,007						-,719	,009		,658	,58	18,849
		(1,552)	(-,571)						(-1,712)	(,609)		,459		(,595)
10	Unconditional CAPM	,005		,036								,408	,89	34,715
		(,376)		(2,216)								,381		(,055)
11	Conditional CAPM	-,004		,039					-,873		,019	,607	,66	15,023
		(-,222)		(1,920)					(-2,260)		(,424)	,548		(,822)
12	Fama-French (3 factors)	-,005		,036	,013	,025						,744	,57	25,202
		(-,398)		(2,188)	(,846)	(1,847)						,682		(,239)
13	Fama-French (5 factors)	-,002		,034	,012	,020	,024	,022				,797	,46	21,056
		(-,171)		(2,013)	(,819)	(1,595)	(2,741)	(1,539)				,685		(,334)
14	*q*-model	,001		,031	,015		,027	,033				,766	,49	24,713
		(,057)		(1,801)	(1,009)		(2,943)	(2,300)				,626		(,213)
Panel C: Monthly data														
15	Unconditional CCAPM	,009	-,050									,364	,26	39,850
		(2,885)	(-2,905)									,302		(,016)
16	Conditional CCAPM	,009	-,089									,475	,24	33,120
		(2,849)	(-3,458)									,389		(,079)
17	Unconditional CAPM	,000		,013								,324	,27	34,338
		(-,077)		(2,346)								,291		(,060)
18	Conditional CAPM	-,003		,015					-,839		,012	,457	,23	18,775
		(-,480)		(2,113)					(-2,052)		(,752)	,356		(,601)
19	Fama-French (3 factors)	-,001		,011	,003	,008						,665	,18	26,310
		(-,123)		(1,958)	(,752)	(1,640)						,629		(,195)
20	Fama-French (5 factors)	,001		,010	,005	,008	,005	-,002				,704	,17	19,445
		(,224)		(1,722)	(1,095)	(1,763)	(1,742)	(-,456)				,663		(,429)
21	*q*-model	,000		,010	,005		,007	,008				,621	,20	27,872
		(,020)		(1,819)	(1,197)		(2,410)	(1,450)				,431		(,112)

Notes: We compile monthly series for all stocks listed in the Tokyo Stock Exchange from the Datastream database, for the period from July 1992 to June 2018. Using this data, we form 25 P/CF-DY portfolios. To determine excess returns, we use the three-month Treasury Bill rate for Japan. Depending on the model, we use the consumption growth, the market portfolio or the Fama-French factors-*q* factors as explanatory variables. In models 2, 4, 9, 11, 16 and 18, we scale factors using the CCI as an instrument. We map the two-pass CSR procedure into GMM to estimate all models, assuming a spectral density matrix with zero leads and lags. We use the same spectral density matrix to run the *J*-test. The table displays two rows for each model, where the first row shows the coefficient estimates and the second row the *t*-statistics. For each model, the columns labeled ‘*R*^2^’ shows the adjusted OLS and GLS *R*^2^ statistics, in that order. All *p*-values resulting from the *J*-tests are in parentheses. Coefficients shown in Panel C for the CCAPM are determined using the factor-mimicking portfolio of the model, as defined in Expression (20), in order to transform the coefficients that result from quarterly consumption data into monthly estimates.

Tables 4–6 display two rows for each model, where the first row shows the lambda estimates that result from Expressions (12) and (15), and the second row shows the *t*-statistics. In the case of the CCAPM, we use the factor-mimicking portfolio approach, as defined in Expressions (16), (17), (19) and (20), to transform the quarterly estimates in Panel B into the monthly results shown in Panel C.As usual in the asset pricing literature, Tables 4–6 report the adjusted OLS *R*^2^ statistics for each model. In any case [67], show that *R*^2^ statistics can give rise to large differences in model performance that are not statistically significant. In fact, the estimated nature of betas can lead to significant distortions in this indicator. In this regard [55], suggest complementing the OLS *R*^2^ statistic with the GLS *R*^2^ statistic, as the latter constitutes a stronger hurdle for asset pricing models. In particular, the authors explain that the GLS *R*^2^ statistic is directly determined by the distance of the model’s factor-mimicking portfolio to the mean-variance frontier, which ultimately conditions the size of the pricing errors provided by the model. Conversely, the OLS *R*^2^ statistic is weakly related to the mean-variance efficiency of factors, leading to spurious conclusions in some cases. Accordingly, Tables 4–6 show both the OLS and GLS *R*^2^ statistics for each model.

**Fig 1:**
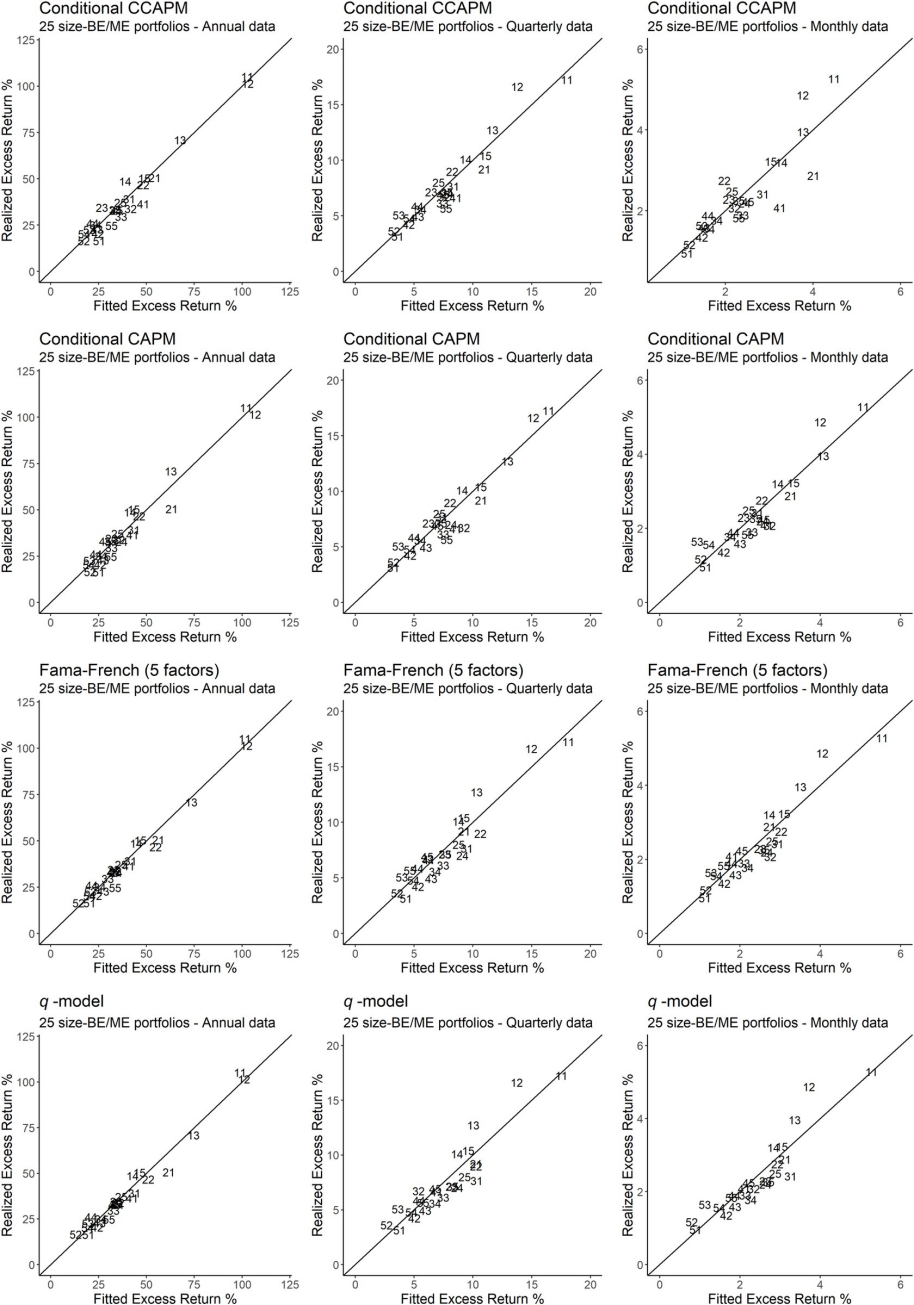
Realized excess returns versus fitted values for 25 portfolios size-BE/ME. The figure depicts 25 portfolios size-BE/ME using a code with two numbers, the first number being the size code (with 1 being the smallest and 5 the largest) and the second number being the BE/ME ratio code (with 1 representing a low ratio and 5 a high ratio). The closer the portfolios are to the 45 degrees axis, the better the fit produced by the model.

**Fig 2:**
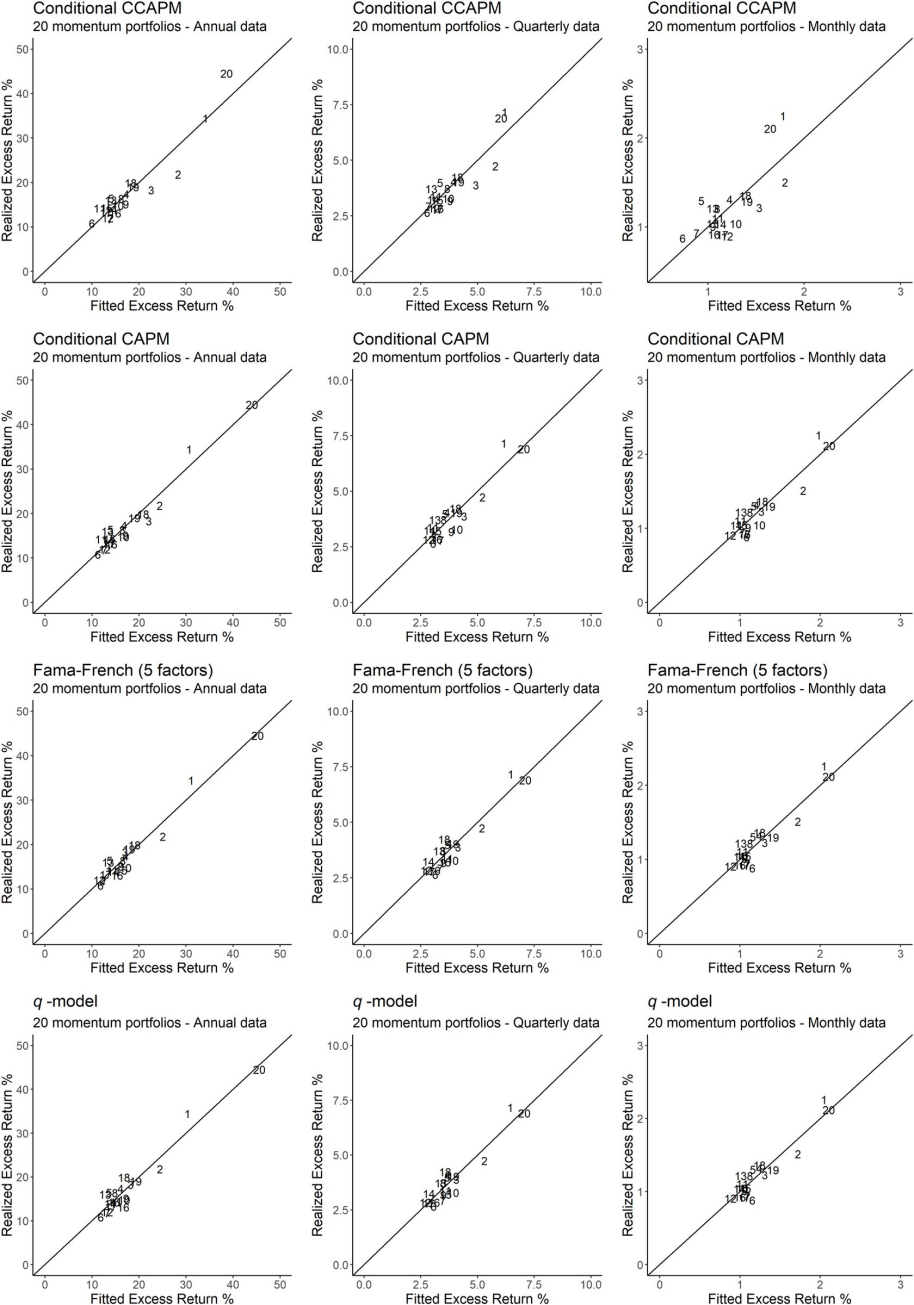
Realized excess returns versus fitted values for 20 momentum portfolios. The figure depicts 20 momentum portfolios using a number from 1 to 20. Stocks with the lowest past one-year return comprise portfolio 1 and stocks with the highest past one-year return comprise portfolio 20. The closer the portfolios are to the 45 degrees axis, the better the fit produced by the model.

**Fig 3:**
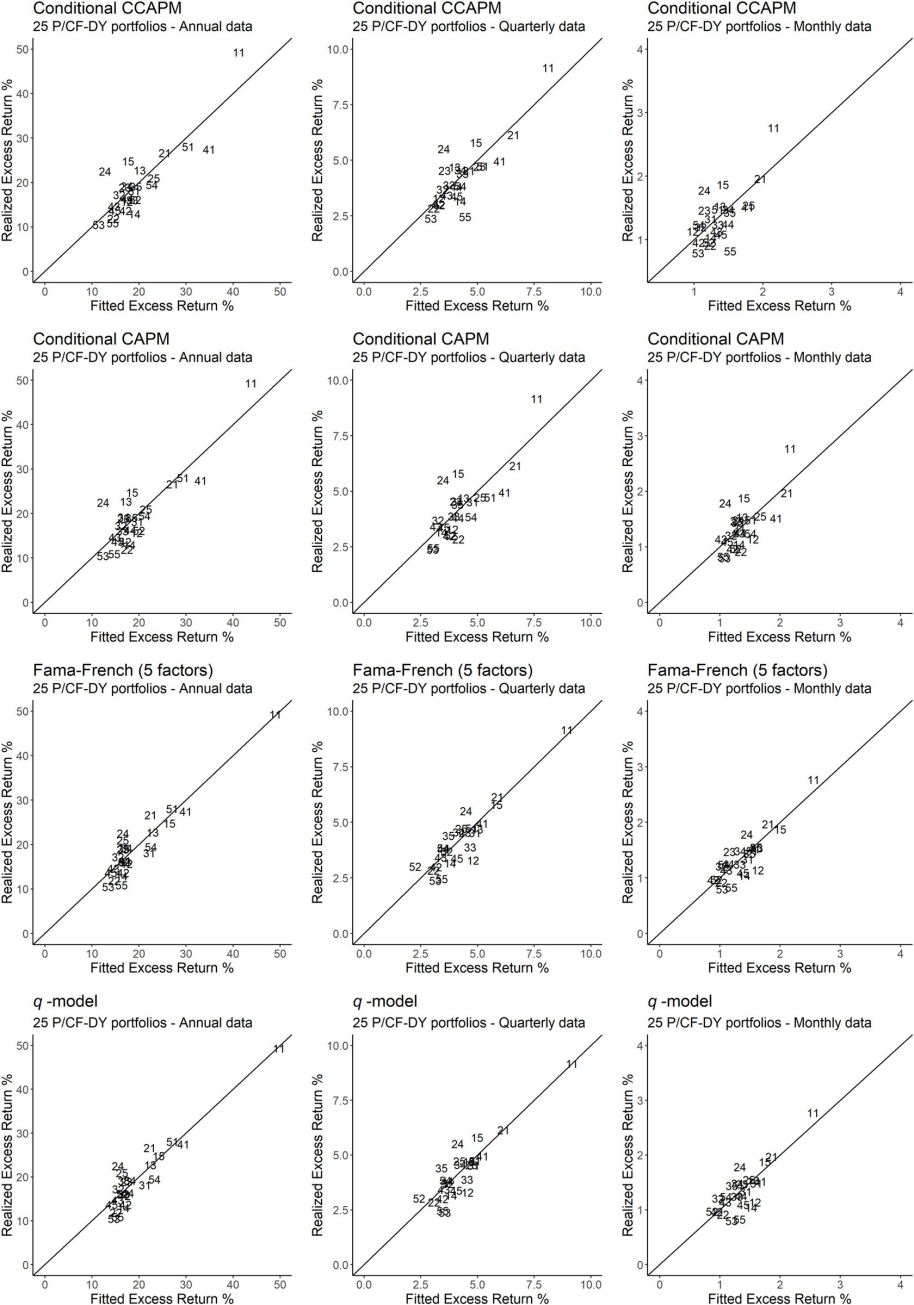
Realized excess returns versus fitted values for 25 portfolios P/CF-DY. The figure depicts 25 portfolios P/CF-DY using a code with two numbers, the first number being the P/CF ratio code (with 1 representing a low ratio and 5 a high ratio) and the second number being the DY code (with 1 representing a low ratio and 5 a high ratio). The closer the portfolios are to the 45 degrees axis, the better the fit produced by the model.

Additionally, problems tied to OLS *R*^2^ statistics and other test statistics make the recent asset pricing literature emphasize the convenience of supplementing these indicators with visual representations of pricing errors. Consequently, Figs 1–3 explicitly relate the fitted values provided by the main models under study to the mean excess returns of the portfolios that constitute our test assets. In particular, any model that produces a perfect fit will provide estimates lying on the 45 degrees angle. Therefore, the better the performance of the model, the more concentrated the estimates will be around the 45 degrees angle, and vice versa. Fig 1 plots the estimates that result from size-BE/ME portfolios, while Figs 2 and 3 do the same for momentum portfolios and P/CF-DY portfolios, respectively.

Tables 4–6 also show the mean absolute pricing error (MAE) for each model, where pricing errors are determined as the difference between the mean excess returns of the portfolios under consideration and the results provided by Expressions (12) and (15), as appropriate. The last column in Tables 4–6 comprises the results delivered by the *J*-test for overidentifying restrictions, as defined by [65].

## Discussion and conclusions

The results shown in Tables 4–6 provide some patterns that unambiguously persist across assets and data frequencies under study. In particular, all results suggest that the CCI helps to strongly improve the performance of single-factor consumption models, namely, the CCAPM and the CAPM. Regarding production models, although the Fama-French five-factor model and the *q*-model are the best-performing models, in most cases the Fama-French five-factor model slightly outperforms the *q*-model. Finally, for all portfolios under study, the factor-mimicking portfolio approach allows the CCAPM to provide monthly results completely consistent with the results obtained at other frequencies. We analyze all results in greater detail below.

Regarding size-BE/ME portfolios, Table 4 shows that use of the CCI as an instrument in the CCAPM and the CAPM allows these models to significantly improve their performance for all the frequencies considered. Specifically, Panel A in Table 4 shows that the CCI allows the CCAPM to increase the OLS *R*^2^ statistic from 0.5 to 0.948, while the GLS *R*^2^ statistic increases from 0.363 to 0.462. Analogously, the OLS *R*^2^ statistic (GLS *R*^2^ statistic) provided by the CAPM rises from 0.542 to 0.944 (0.359 to 0.611) when the CCI is used as an instrument. Consistently, for these models, the MAE is more than halved in the conditional versions with respect to their unconditional counterparts. This pattern persists in Panels B and C, using quarterly and monthly return data, respectively. The case of the CAPM in Panel C is especially remarkable, as the unconditional model provides an OLS *R*^2^ statistic and a MAE of 0.145 and 0.75%, respectively, while its conditional counterpart provides estimates of 0.86 and 0.3% for the same statistics.

Nevertheless, it should be noted that the conditional models under study only account for the conditioning information captured by the CCI, while information sets that investors use to drive their economic decisions comprise a large number of variables, where most of them are unobservable. Although this fact is intrinsic to any asset pricing model, to observe to what extent the explanatory power of the CCI persists when other macroeconomic variables are considered, Tables A1–A3 in the S1 Appendix show the regression results of the conditional models, using both Δ*GDP* and the CPI as additional explanatory variables. As shown in Table A1 in S1 Appendix, in most cases, the results remain almost unchanged from those shown in Table 4 when these variables are included, with few exceptions, suggesting that, in general, Δ*GDP* and the CPI hardly contribute to improve the explanatory power of the model.

Table 4 shows that the conditional versions of both the CCAPM and the CAPM perform very similarly to the Fama-French five-factor model and the *q*-model. However, in most cases, the Fama-French five-factor model is the best-performing model followed by the *q*-model, providing OLS and GLS *R*^2^ statistics greater than 0.9 in Panel A and around 0.8 in Panels B and C. Furthermore, the GLS *R*^2^ statistic magnifies the differences between single-factor consumption models and production models. Specifically, although all GLS *R*^2^ statistics are lower than OLS *R*^2^ statistics, they decline more markedly in the case of the CCAPM and the CAPM, meaning that their factor-mimicking portfolios are further from the mean-variance frontier than those of production models.

Remarkably, the *J*-test for overidentifying restrictions rejects most models under analysis, with the exception of the CCAPM, the CAPM and the Fama-French five-factor model in Panel B (rows 8–11 and 13), and the conditional CAPM and the Fama-French five-factor model in Panel C (rows 18 and 20).

Although the coefficient estimates shown in Table 4, Panel C, for the Fama-French five-factor model and the *q*-model do not exactly coincide with the means of their respective factors (see Table 1, Panel D), the prices of risk for RMRF, SMB and *r*_*I*/*A*_ in the *q*-model are relatively close to their average returns, meaning that the model is reasonably well-behaved. Conversely, the CAPM provides estimates that are far from the mean return of RMRF, which means that the model does not correctly price the market portfolio.

Fig 1 shows that the fitted values provided by all the models under consideration are strongly concentrated around the 45 degrees axis, which is consistent with the results shown in Table 4.

For momentum portfolios, the results shown in Table 5 are fully in line with those shown in Table 4 for size-BE/ME portfolios. Notably, the unconditional CAPM performs satisfactorily in pricing momentum portfolios (rows 3, 10 and 17), although the use of the CCI as an instrument significantly improves its performance. Specifically, the OLS *R*^2^ statistic provided by the conditional version of the model amounts to 0.935, 0.834 and 0.826, using annual, quarterly and monthly data, respectively, while the GLS *R*^2^ statistics amount to 0.864, 0.818 and 0.809 for the same frequencies. Nonetheless, the CCI has an even greater impact on the CCAPM. Indeed, as shown in Table 5, Panel B, the unconditional CCAPM delivers an OLS *R*^2^ statistic and a MAE of 0.192 and 0.75%, respectively, while its conditional counterpart leads to values of 0.755 and 0.42%, for the same statistics. Furthermore, results in Panel C show that the positive effect of the CCI on the performance of the CCAPM persists using monthly data. In particular, rows 15 and 16 in Table 5 show that the OLS *R*^2^ statistic (GLS *R*^2^ statistic) increases from 0.168 to 0.602 (0.09 to 0.574) when we use the CCI as an instrument. Table A2 in the S1 Appendix shows that the results of conditional models barely vary when Δ*GDP* and the CPI are used as additional explanatory variables, which corroborates the informativeness of the CCI.

As with size-BE/ME portfolios, for momentum portfolios, the Fama-French five-factor model and the *q*-model are generally the best-performing models, although the Fama-French three-factor model also provides excellent results. Notably, the results of both the Fama-French five-factor model and the *q*-model are even closer in Table 5 than in Table 4. In fact, in Table 5, Panels B and C, their MAEs are exactly the same, amounting to 0.35% and 0.11%, respectively.

Unlike size-BE/ME portfolios studied in Table 4, for momentum portfolios, the *J*-test for overidentifying restrictions does not reject most of the models in Panels B and C, with the sole exception of the unconditional CCAPM in row 15. However, Panel A shows that the opposite is true on an annual basis, with the CCAPM being the only model not rejected by the *J*-test.

Again, the coefficient estimates shown in Table 5, Panel C, for the Fama-French five-factor model and the *q*-model do not coincide with the means of the factors. However, in both models, the prices of risk for *r*_*I*/*A*_ and *r*_*ROE*_ are very close to their average returns, which means that these models correctly price both investment and profitability factors.

Fig 2 shows that the vast majority of the models considered produce low pricing errors for momentum portfolios, with the conditional CCAPM being the worst-performing model when used on monthly data, consistently with the results shown in Table 5.

Regarding portfolios P/CF-DY, Table 6 shows that, in general, all models into consideration perform worse than in the other samples. Table 6 also shows that, in this case, the CCI helps consumption models to improve their performance to a lesser extent than in Tables 4 and 5. In fact, although the conditional versions of both the CCAPM and the CAPM provide higher OLS *R*^2^ statistics than their unconditional counterparts, the GLS *R*^2^ statistics shown in Panel A for these models decrease when we use the CCI as an instrument, even though their MAEs drop. This suggests that both model factors and consumer sentiment are less explanatory for P/CF-DY portfolios than for the other anomaly portfolios considered. Remarkably, Table A3 in the S1 Appendix shows that neither Δ*GDP* nor the CPI help to improve the performance of the conditional models.

Notably, results shown in Table 6 widen the gap between the Fama-French five-factor model and the *q*-model, with the latter performing significantly worse than the former. The case of Panel C in Table 6 is especially remarkably, as the *q*-model provides lower OLS and GLS *R*^2^ statistics and a higher MAE than both the Fama-French three- and five-factor models. As in previous tables, Table 6 shows that the Fama-French five-factor model generally provides the highest OLS and GLS *R*^2^ statistics and the lowest MAEs among the models under study.

Although the *J*-test for overidentifying restrictions does not reject most of the models in Panels B and C, this may be partly explained by the high variances and covariances in the spectral density matrix, rather than the low pricing errors provided by these models.

It is important noting that lambda estimates shown in Panel C for the Fama-French five-factor model and the *q*-model are close to the mean of their respective factors for RMRF, *r*_*I*/*A*_ and *r*_*ROE*_ (see Table 1, Panel D). Additionally, the prices of risk that result from the conditional and unconditional CAPM in Panel C are relatively close to the mean of RMRF, meaning that these models are reasonably well-behaved.

Fig 3 shows that all fitted values provided by the models under consideration are more scattered than those depicted in Figs 1 and 3, which is consistent with the results shown in Table 6.

In summary, the results shown in Tables 4–6 provide us with three major findings. First, in most cases, the CCI does an excellent job when used as an instrument in single-factor consumption models, contributing to significantly improve their performance for most frequencies and portfolios under study. Second, the Fama-French five-factor model and the *q*-model are the best-performing models among those considered in the research. However, in the vast majority of cases the Fama-French five-factor model slightly outperforms the *q*-model, which means that HML and investment factors do not appear to be completely redundant on the Tokyo Stock Exchange. Third, the results provided by the CCAPM using monthly market data are fully consistent with those delivered by the model at other frequencies, which suggests that the model’s factor-mimicking portfolio does a good job of retaining the pricing information included in consumption growth.

Most of the asset pricing models under consideration perform remarkably well for both size-BE/ME portfolios and momentum portfolios, while performing worse for P/CF-DY portfolios. Regarding the periodicity of the data, in all cases, the higher the frequency of the data, the worse the performance of the models, which is particularly evident in the case of the CCAPM and the CAPM.

In the light of these results, future research should deepen the study on the connection between consumption and production asset pricing models. Indeed, both approaches constitute partial solutions in the analysis of the relationship between asset prices and macroeconomics, assuming that demand or supply are determined exogenously. In contrast, full general equilibrium models with production use technology shocks as the primary driving process of macroeconomic uncertainty. The fact that the CCI contributes strongly to improving the performance of classic consumption-based asset pricing models can help strengthen the interplay between consumption and production models. In this regard, while [22] and [23] emphasize the relationship between the predictive power of the CCI and habit formation [68–70], embed habit-formation preferences within production models. This makes consumer sentiment a promising tool for relating consumption and production models more explicitly.

Additionally, further research should explore the sensitivity of the model estimates to different estimation procedures. In this regard, the methodology suggested by [66] and [32–34] can be a promising tool to address endogeneity and other specification issues related to the two-pass CSR estimates.

Nevertheless, it is worth noting that none of the models studied in this paper allows us to perfectly explain the expected returns for all portfolios and frequencies under analysis, which makes us consider other explanations for asset prices apart from those arising from a purely macroeconomic perspective. Indeed, pricing errors are directly related to the joint hypothesis problem, that is, the fact that tests on the efficiency of financial markets are completely equivalent to tests of economic discount factor models, which implies that market efficiency is only testable in combination with a model of expected returns [17] (p. 122). In this regard, the fact that most statistical tests on the efficiency of the Tokyo Stock Exchange show mixed results [71–73] opens the door to behavioral explanations for the fraction of expected returns that remains unexplained on the Japanese equity market. Particularly, partial equilibrium models, such as those based on heterogeneous investors, can help to successfully complement this view [33, 74–79].

## Supporting information

S1 Data Portfolio returns and macroeconomic series. (PDF)

S1 Appendix Regression results using additional macroeconomic factors. (DOCX)

S1 File (TXT)

S2 File (TXT)

S3 File (TXT)

S4 File (TXT)

S5 File (TXT)

S6 File (TXT)

S7 File (TXT)

S8 File (TXT)

S9 File (TXT)

S10 File (TXT)

## References

[1] Cochrane JH. Presidential address: Discount rates. J Finance. 2011;66(4):1047–108.

[2] Lettau M, Ludvigson S. Resurrecting the (c)CAPM: A cross-sectional test when risk premia are time-varying. J Polit Econ. 2001;109(6):1238–87.

[3] Lustig H, Van Nieuwerburgh S. Housing collateral, consumption insurance, and risk premia: An empirical perspective. J Finance. 2005;60(3):1167–219.

[4] Sanchez JM, Yurdagul E. A look at japan's slowdown and its turnaround plan. Region Econ. 2014;22(1):5–9.

[5] Anderson D, Botman DP, Hunt BL. Is japan’s population aging deflationary? International Monetary Fund, 2014 Contract No.: 14/139.

[6] Fama EF, French KR. A five-factor asset pricing model. J Financ Econ. 2015;116(1):1–22.

[7] Hou K, Xue C, Zhang L. Digesting anomalies: An investment approach. Rev Financ Stud. 2014;28(3):650–705.

[8] Cochrane JH. Asset pricing (revised edition). New Jersey, United States of America: Princeton University Press; 2005.

[9] Sharpe WF. Capital asset prices: A theory of market equilibrium under conditions of risk. J Finance. 1964;19(3):425–42.

[10] Lintner J. The valuation of risk assets and the selection of risky investments in stock portfolios and capital budgets. Rev Econ Stat. 1965;47(1):13–37.

[11] Lintner J. Security prices, risk, and maximal gains from diversification. J Finance. 1965;20(4):587–615.

[12] Lucas R, E. Asset prices in an exchange economy. Econometrica. 1978;46(6):1429–45.

[13] Breeden DT. An intertemporal asset pricing model with stochastic consumption and investment opportunities. J Financ Econ. 1979;7(3):265–96.

[14] Fama EF, French KR. Common risk factors in the returns on stocks and bonds. J Financ Econ. 1993;33(1):3–56.

[15] Maio P, Santa-Clara P. Multifactor models and their consistency with the ICAPM. J Financ Econ. 2012;106:586–613.

[16] Merton R. An inter-temporal capital asset pricing model. Econometrica. 1973;41:867–87.

[17] Campbell JY. Financial decissions and markets: A course in asset pricing. New Jersey, United States of America: Princeton University Press; 2018.

[18] Lemmon M, Portniaguina E. Consumer confidence and asset prices: Some empirical evidence. Rev Financ Stud. 2006;19(4):1499–529.

[19] Chen S-S. Lack of consumer confidence and stock returns. J Empir Financ. 2011;18:225–36.

[20] Huang D, Jiang F, Tu J, Zhou G. Investor sentiment aligned: A powerful predictor of stock returns. Rev Financ Stud. 2014;28(3):791–837.

[21] Bertella M, Pires F, Feng L, Stanley H. Confidence and the stock market: An agent-based approach. PLOS ONE. 2014;9:e83488. 24421888 10.1371/journal.pone.0083488 PMC3885419

[22] Ludvigson S. Consumer confidence and consumer spending. J Econ Perspect. 2004;18(2):29–50.

[23] Sommer M. Habit formation and aggregate consumption dynamics. BE J Macroecon. 2007;7(1):1–25.

[24] Chua CL, Tsiaplias S. Can consumer sentiment and its components forecast Australian GDP and consumption? J Forecasting. 2009;28(8):698–711.

[25] Song M, Shin K-s. Forecasting economic indicators using a consumer sentiment index: Survey-based versus text-based data. J Forecasting. 2019;38(6):504–18.

[26] Hamao Y. An empirical examination of the arbitrage pricing theory: Using Japanese data. Jpn World Econ. 1988;1(1):45–61.

[27] Azeez AA, Yonezawa Y. Macroeconomic factors and the empirical content of the arbitrage pricing theory in the Japanese stock market. Jpn World Econ. 2006;18(4):568–91.

[28] Zaremba A. Investor sentiment, limits on arbitrage, and the performance of cross-country stock market anomalies. J Behav Exp Econ. 2016;9:136–63.

[29] Baker M, Wurgler J. Investor sentiment and the cross-section of stock returns. J Finance. 2006;61(4):1645–80.

[30] Amihud Y. Illiquidity and stock returns: Cross-section and time-series effects. J Financ Mark. 2002;5(1):31–56.

[31] Pástor L, Stambaugh R. Liquidity risk and expected stock returns. J Polit Econ. 2003;111(3):642–85.

[32] Racicot F-É, Rentz WF, Théoret R. Testing the new Fama and French factors with illiquidity: A panel data investigation. Finance. 2018;39(3):45–102.

[33] Racicot F-É, Rentz WF, Tessier D, Théoret R. The conditional Fama-French model and endogenous illiquidity: A robust instrumental variables test. PLOS ONE. 2019;14(9):1–26. 10.1371/journal.pone.0221599 31532780 PMC6750890

[34] Racicot F-E, Rentz WF, Kahl A, Mesly O. Examining the dynamics of illiquidity risks within the phases of the business cycle. Borsa Istanbul Rev. 2019;19(2):117–31. 10.1016/j.bir.2018.12.001

[35] Fazzari SM, Hubbard RG, Petersen BC. Financing constraints and corporate investment. Brookings Pap Eco Ac. 1988(1):141–206.

[36] Cochrane JH. Production-based asset pricing and the link between stock returns and economic fluctuations. J Finance. 1991;46(1):209–37.

[37] Chirinko R. Business fixed investment spending: Modeling strategies, empirical results, and policy implications. J Econ Lit. 1993;31:1875–911.

[38] Abel AB, Eberly JC. A unified model of investment under uncertainty. Am Econ Rev. 1994;84(5):1369–84.

[39] Cochrane JH. A cross-sectional test of an investment-based asset pricing model. J Polit Econ. 1996;104(3):572–621.

[40] Lamont OA. Investment plans and stock returns. J Finance. 2000;55(6):2719–45.

[41] Campbell JY, Shiller RJ. The dividend-price ratio and expectations of future dividends and discount factors. Rev Financ Stud. 1988;1(3):195–228.

[42] Carhart MM. On persistence in mutual fund performance. J Finance. 1997;52(1):57–82.

[43] Zhang L. The investment CAPM. Eur Financ Manag. 2017;23(4):545–603.

[44] Ross SA. A simple approach to the valuation of risky streams. J Bus. 1978;51(3):453–75.

[45] Harrison JM, Kreps DM. Martingales and arbitrage in multiperiod securities markets. J Econ Theory. 1979;20(3):381–408.

[46] Hansen LP, Richard SF. The role of conditioning information in deducing testable restrictions implied by dynamic asset pricing models. Econometrica. 1987;55(3):587–613.

[47] Ross S. The arbitrage theory of capital asset pricing. J Econ Theory. 1976;13(3):341–60.

[48] Roll R, Ross SA. An empirical investigation of the arbitrage pricing theory. J Finance. 1980;35(5):1073–103.

[49] Tsiaplias S. Factor estimation using MCMC-based Kalman filter methods. Comput Stat Data An. 2008;53(2):344–53.

[50] Ferson WE, Schadt RW. Measuring fund strategy and performance in changing economic conditions. J Finance. 1996;51(2):425–61.

[51] Ghosh A, Julliard C, Taylor AP. What is the consumption-CAPM missing? An information-theoretic framework for the analysis of asset pricing models. Rev Financ Stud. 2016;30(2):442–504.

[52] Fama EF, French KR. International tests of a five-factor asset pricing model. J Financ Econ. 2017;123(3):441–63.

[53] Rojo-Suárez J, Alonso-Conde AB. Data for: Impact of consumer confidence on the expected returns of the tokyo stock exchange: A comparative analysis of consumption and production-based asset pricing models 2020. Available from: 10.17632/vyxt842rzg.2. PMC7608913 33141827

[54] Griffin JM, Kelly P, Nardari F. Do market efficiency measures yield correct inferences? A comparison of developed and emerging markets. Rev Financ Stud. 2010;23(8):3225–77.

[55] Lewellen J, Nagel S, Shanken J. A skeptical appraisal of asset pricing tests. J Financ Econ. 2010;96(2):175–94.

[56] Parker J, A., Julliard C. Consumption risk and the cross section of expected returns. J Polit Econ. 2005;113(1):185–222.

[57] Ferson WE, Sarkissian S, Simin TT. Spurious regressions in financial economics? J Finance. 2003;58(4):1393–413.

[58] Hansen LP, Hodrick RJ. Forward exchange rates as optimal predictors of future spot rates: An econometric analysis. J Polit Econ. 1980;88(5):829–53.

[59] Black FS, Jensen MC, Scholes MS. The capital asset pricing model: Some empirical findings In: Jensen MC, editor.: Praeger; 1972 p. 79–124.

[60] Fama E, MacBeth JD. Risk, return, and equilibrium: Empirical tests. J Polit Econ. 1973;81(3):607–36.

[61] Pagan A. Econometric issues in the analysis of regressions with generated regressors. Int Econ Rev. 1984;25(1):221–47.

[62] Pagan A. Two stage and related estimators and their applications. Rev Econ Stud. 1986;53(4):517–38.

[63] Pagan A, Ullah A. The econometric analysis of models with risk terms. J Appl Econom. 1988;3(2):87–105.

[64] Shanken J. On the estimation of beta-pricing models. Rev Financ Stud. 1992;5(1):1–33.

[65] Hansen LP. Large sample properties of generalized method of moments estimators. Econometrica. 1982;50(4):1029–54.

[66] Racicot F-É. Engineering robust instruments for GMM estimation of panel data regression models with errors in variables: A note. Appl Econ. 2015;47(10):981–9.

[67] Kan R, Robotti C, Shanken J. Pricing model performance and the two-pass cross-sectional regression methodology. J Finance. 2013;68(6):2617–49.

[68] Jermann UJ. Asset pricing in production economies. J Monetary Econ. 1998;41(2):257–75.

[69] Boldrin M, Christiano LJ, Fisher JDM. Habit persistence, asset returns, and the business cycle. Am Econ Rev. 2001;91(1):149–66.

[70] Melino A, Yang A. State dependent preferences can explain the equity premium puzzle. Rev Econ Dynam. 2003;6:806–30.

[71] Chan KC, Gup BE, Pan M-S. International stock market efficiency and integration: A study of eighteen nations. J Bus Finan Account. 1997;24(6):803–13.

[72] Sohel Azad ASM. Efficiency, cointegration and contagion in equity markets: Evidence from China, Japan and South Korea*. Asian Econ J. 2009;23(1):93–118.

[73] Seth N, Sharma AK. International stock market efficiency and integration: Evidences from Asian and us markets. J Adv Manag Res. 2015;12(2):88–106.

[74] Navarro RM, Larralde H. A detailed heterogeneous agent model for a single asset financial market with trading via an order book. PLOS ONE. 2017;12(2):1–27. 10.1371/journal.pone.0170766 PMC5330465 28245251

[75] Campbell JY. Household finance. J Finance. 2006;61(4):1553–604.

[76] Farhi E, Panageas S. Saving and investing for early retirement: A theoretical analysis. J Financ Econ. 2007;83:87–121.

[77] Kocherlakota N, Pistaferri L. Asset pricing implications of pareto optimality with private information. J Polit Econ. 2009;117(3):555–90.

[78] Guvenen F. A parsimonious macroeconomic model for asset pricing. Econometrica. 2009;77(6):1711–50.

[79] Duffie D. Presidential address: Asset price dynamics with slow-moving capital. J Finance. 2010;65(4):1237–67.

