# Supplementary material for: Impact of consumer confidence on the expected returns of the Tokyo Stock Exchange: A comparative analysis of consumption and production-based asset pricing models
Source: PLoS One. 2020 Nov 3;15(11):e0241318. doi: 10.1371/journal.pone.0241318 (PMC7608913; doi:10.1371/journal.pone.0241318)
Supplement: S1 Data — (PDF) [file pone.0241318.s001.pdf]

Data for: Impact of consumer confidence on the expected returns of the Tokyo Stock Exchange: A comparative analysis of consumption and production-based asset pricing models

Using all stocks listed in the Tokyo Stock Exchange and macroeconomic data for Japan, the dataset comprises the following series:

1. Monthly returns for 25 size-book-to-market equity portfolios, following the Fama and French (1993) methodology. (Raw data source: Datastream database)
2. Monthly returns for 20 momentum portfolios, following the Fama and French (1993) methodology. (Raw data source: Datastream database)
3. Monthly returns for 25 price-to-cash flow-dividend yield portfolios, following the Fama and French (1993) methodology. (Raw data source: Datastream database)
4. Fama and French three-factors (RM, SMB and HML), following the Fama and French (1993) methodology. (Raw data source: Datastream database)
5. Fama and French five-factors (RM, SMB, HML, RMW, and CMA), following the Fama and French (2015) methodology for all factors, except for RMW, which is determined using the return on assets as sorting variable, as in Hou, Xue and Zhang (2014). (Raw data source: Datastream database)
6. Private final consumption expenditure, in national currency and constant prices, non-seasonally adjusted, for Japan. (Raw data source: OECD)
7. Consumer Confidence Index (CCI) for Japan. (Raw data source: OECD)
8. Three-month interest rate of the Treasury Bill for Japan. (Raw data source: OECD)
9. Gross Domestic Product (GDP) for Japan. (Raw data source: OECD)
10. Consumer Price Index (CPI) growth rate for Japan. (Raw data source: OECD)

We have produced all return series using the following data from Datastream: (i) total return index (RI series), (ii) market value (MV series), (iii) market-to-book equity (PTBV series), (iv) total assets (WC02999 series), (v) return on equity (WC08301 series), (vi) price-to-cash flow ratio (PC series), and (vii) dividend yield (DY series). We have used the generic rules suggested by Griffin, Kelly, & Nardari (2010) for excluding non-common equity securities from Datastream data. We also exclude stocks with less than twelve observations in the period from July 1992 to June 2018. Accordingly, our sample comprises a total number of 5,312 stocks.

References:

Fama, E. F. and French, K. R. (1993). Common risk factors in the returns on stocks and bonds. Journal of Financial Economics, 33, 3–56.

Fama, E. F. and French, K. R. (2015). A five-factor asset pricing model. Journal of Financial Economics, 116, 1–22.

Griffin, J. M., Kelly, P., and Nardari, F. (2010). Do market efficiency measures yield correct inferences? A comparison of developed and emerging markets. Review of Financial Studies, 23, 3225–3277.

Hou K, Xue C, Zhang L. (2014). Digesting anomalies: An investment approach. Review of Financial Studies, 28, 650-705.
