# Supplementary material for: Impact of consumer confidence on the expected returns of the Tokyo Stock Exchange: A comparative analysis of consumption and production-based asset pricing models
Source: PLoS One. 2020 Nov 3;15(11):e0241318. doi: 10.1371/journal.pone.0241318 (PMC7608913; doi:10.1371/journal.pone.0241318)
Supplement: S1 Appendix — (DOCX) [file pone.0241318.s002.docx]

**Appendix**

**Table A1. Regression results for 25 portfolios size-BE/ME**.

Notes: We compile monthly series for all stocks listed in the Tokyo Stock Exchange from the Datastream database, for the period from July 1992 to June 2018. Using this data, we form 25 size-BE/ME portfolios. To determine excess returns, we use the three-month Treasury Bill rate for Japan. Depending on the model, we use the consumption growth, the market portfolio, the GDP growth rate or the CPI as explanatory variables. Additionally, we scale consumption growth or RMRF using the CCI as an instrument. We map the two-pass CSR procedure into GMM to estimate all models, assuming a spectral density matrix with zero leads and lags. We use the same spectral density matrix to run the *J*-test. The table displays two rows for each model, where the first row shows the coefficient estimates and the second row the *t*-statistics. For each model, the columns labeled ‘$R^{2}$’ shows the adjusted OLS and GLS $R^{2}$ statistics, in that order. All *p*-values resulting from the *J*-tests are in parentheses. Coefficients shown in Panel C are determined using the factor-mimicking portfolio of the models, as defined in Expression (20), in order to transform the coefficients that result from quarterly macroeconomic data into monthly estimates.

**Table A2. Regression results for 20 momentum portfolios**.

Notes: We compile monthly series for all stocks listed in the Tokyo Stock Exchange from the Datastream database, for the period from July 1992 to June 2018. Using this data, we form 20 momentum portfolios. To determine excess returns, we use the three-month Treasury Bill rate for Japan. Depending on the model, we use the consumption growth, the market portfolio, the GDP growth rate or the CPI as explanatory variables. Additionally, we scale consumption growth or RMRF using the CCI as an instrument. We map the two-pass CSR procedure into GMM to estimate all models, assuming a spectral density matrix with zero leads and lags. We use the same spectral density matrix to run the J-test. The table displays two rows for each model, where the first row shows the coefficient estimates and the second row the t-statistics. For each model, the columns labeled ‘R^2’ shows the adjusted OLS and GLS R^2 statistics, in that order. All p-values resulting from the J-tests are in parentheses. Coefficients shown in Panel C are determined using the factor-mimicking portfolio of the models, as defined in Expression (20), in order to transform the coefficients that result from quarterly macroeconomic data into monthly estimates.

**Table A3. Regression results for 25 portfolios P/CF-DY.**

Notes: We compile monthly series for all stocks listed in the Tokyo Stock Exchange from the Datastream database, for the period from July 1992 to June 2018. Using this data, we form 25 P/CF-DY portfolios. To determine excess returns, we use the three-month Treasury Bill rate for Japan. Depending on the model, we use the consumption growth, the market portfolio, the GDP growth rate or the CPI as explanatory variables. Additionally, we scale consumption growth or RMRF using the CCI as an instrument. We map the two-pass CSR procedure into GMM to estimate all models, assuming a spectral density matrix with zero leads and lags. We use the same spectral density matrix to run the *J*-test. The table displays two rows for each model, where the first row shows the coefficient estimates and the second row the *t*-statistics. For each model, the columns labeled ‘$R^{2}$’ shows the adjusted OLS and GLS $R^{2}$ statistics, in that order. All *p*-values resulting from the *J*-tests are in parentheses. Coefficients shown in Panel C are determined using the factor-mimicking portfolio of the models, as defined in Expression (20), in order to transform the coefficients that result from quarterly macroeconomic data into monthly estimates.
